# Economic Evaluations of New Vaccine Introduction in Middle-Income Countries in the Middle East and North Africa Region: A Systematic Review

**DOI:** 10.3390/vaccines14070591

**Published:** 2026-07-02

**Authors:** Chrissy Bishop, Konstantina Politopoulou, Maria Bermudez, Federico Rodriguez-Cairoli, Motuma Abeshu, Sowmya Kadandale, Ibironke Oyatoye, Saadia Farrukh

**Affiliations:** 1 Triangulate Health Ltd., Doncaster DN11 9QU, UK; 2 UNICEF MENA Regional Office, Abdulqader Al-Abed Street, Building No. 15 Tla’a Al-Ali, Amman 11821, Jordan; 3 UNICEF Headquarters, 3 UN Plaza, Room 816, New York, NY 10017, USA

**Keywords:** economic evaluation, Middle East and North Africa, vaccines

## Abstract

Background/Objectives: Middle-income countries (MICs) in the Middle East and North Africa (MENA) face financial and health system barriers when introducing new vaccines. The Gavi MICs approach has supported the introduction of pneumococcal conjugate (PCV), human papillomavirus (HPV), and rotavirus (RV) vaccines; however, economic evidence from the region remains limited. This systematic review assessed the quantity, characteristics, and quality of economic evaluations of these vaccines in MENA MICs published between 2015 and 2025 and synthesised economic evidence to inform policy decisions in Algeria, Egypt, Iran, Jordan, Lebanon, Morocco, Palestine, and Tunisia. Methods: Relevant databases and registries were searched for cost–effectiveness, cost–utility, cost–benefit, and budget impact analyses of PCV, HPV, and RV vaccination strategies. Two reviewers independently screened studies, extracted data, and assessed methodological quality. Results: Twenty-six studies met the inclusion criteria, including 12 on HPV, nine on RV, and five on PCV. Vaccine introduction was the most commonly evaluated intervention (*n* = 23), and most studies were cost–effectiveness or cost–utility analyses adopting payer, health system, societal, or mixed perspectives. PCV and RV introduction were consistently found to be cost-effective or cost-saving. HPV introduction showed mixed results, particularly in Iran, but was generally cost-effective in Tunisia and Morocco. Reporting of vaccine coverage, delivery costs, and programmatic constraints was limited, and overall methodological quality varied. Conclusions: Available evidence supports the economic value of PCV and RV introduction in MENA MICs, while HPV’s cost-effectiveness is context dependent. Future evaluations should incorporate dynamic modelling, implementation costs, and affordability considerations to better inform sustainable vaccine introduction.

## 1. Introduction

Vaccine introduction has been challenging in the Middle-East and North Africa (MENA) region’s middle-income countries (MICs). Following the World Bank’s 2024 income classification, MICs are economies with a gross national income (GNI) per capita, calculated using the World Bank Atlas method, of between $1136 and $4495 (lower middle-income) and between $4496 and $13,935 (upper middle-income) [1]. These countries face high vaccine costs alongside fiscal pressures and disruptions from conflict and displacement [2,3]. To address these barriers, the Gavi MICs approach (5.0) provides catalytic tools to support the sustainable introduction of the human papillomavirus vaccine (HPV), the pneumococcal conjugate vaccine (PCV) and the rotavirus vaccine (RV) in eligible MENA MICs, which, at the time of writing, included Algeria, Egypt, Iran, Jordan, Lebanon, Morocco, the State of Palestine, and Tunisia [4]. These three vaccines were selected as the focus of this review because they are the vaccines prioritised under the Gavi MICs 5.0 framework for the region.

In low- and middle-income countries (LMICs), HPV vaccination in girls could reduce cervical cancer incidence by over 89% and avert 61 million cases over the next century [5]. PCVs demonstrate 54% effectiveness against severe pneumonia in children under five [6]. RVs can reduce severe rotavirus diarrhoea by 82–92% in low-mortality countries, and 35–63% in high-mortality countries [7]. Despite the benefits of these vaccines, their rollout remains uneven in the MENA region [8,9,10]. Morocco is the only MIC in the region that has introduced all three vaccines, while Egypt has yet to add any to its national programme [11]. Other MICs have introduced PCVs and/or RVs, and while Tunisia introduced HPVs in 2025, but the broader introduction of HPV continues to face delays [11]. One of the most critical barriers is the financial feasibility of new vaccine introduction, compounded by declining government expenditure on vaccines and political and economic instability [11]. In such resource-constrained settings, economic evaluations are key decision-making tools, as they compare the costs and health outcomes of competing interventions to identify the best value for money. This is especially important for vaccine introduction, where large upfront and recurrent costs must be weighed against long-term health gains and sustained domestic financing.

The evidence base supporting vaccine introduction in the region is limited. A 2021 systematic review identified 46 economic evaluations of vaccines in the MENA region conducted up to 2019 [12]. In these studies, the most commonly assessed vaccines were RV (33%), HPV (17%) and PCV (15%). However, evidence was concentrated in a small number of countries, such as Iran, Israel, and Turkey [12]. Study quality was also variable, with only a small proportion rated ‘excellent’, while the majority were assessed as ‘good’ or ‘moderate’ [12]. No similar systematic reviews of new vaccines in the MENA region were found after 2021, yet the evidence base has expanded, with several new economic evaluations published in the region [13,14,15,16]. Further, Nagi et al. (2021) [12] only covered studies published up to 2019, making their evidence base now five years out of date. As a result, there is no consolidated and up-to-date evidence base to support national decision-making on vaccine introduction and financing in the MENA region [12].

This systematic review aims to assess the quantity, characteristics, and quality of economic evaluations conducted in MENA MICs eligible under the Gavi 5.0 framework and published during the 2015–2025 period, focusing on three Gavi-prioritised vaccines (PCV, HPV, RV). This review is one component of a wider UNICEF MENA Regional Office programme. Its role is diagnostic, mapping where economic evaluations are absent or methodologically limited to target subsequent efforts to inform policymaking on vaccine uptake. The objectives were to:

- Identify economic evaluations that assess the costs and benefits of introducing HPVs, PCVs, and RVs into National Immunisation Plans in MENA MICs eligible under the Gavi 5.0 framework.
- Synthesise existing economic evaluations of HPVs, PCVs, and RVs to inform policymaking on the economic feasibility and sustainability of vaccine uptake.
- Highlight evidence gaps and priority areas for further research.

## 2. Materials and Methods

### 2.1. Protocol and Registration

The review protocol was prospectively registered with the International Prospective Register of Systematic Reviews (PROSPERO registration number CRD420251234865; this can be accessed here https://www.crd.york.ac.uk/PROSPERO/search (accessed on 23 June 2026)). The systematic literature review was conducted in accordance with the PRISMA 2020 checklist [17]. Methods were guided by the Cochrane Handbook for Systematic Reviews of Interventions (v6.4).

### 2.2. Data Sources and Searches

A comprehensive literature search was conducted in MEDLINE via PubMed, the Tufts Cost-Effectiveness Analysis (CEA) Registry, and the International Health Technology Assessment (INAHTA) Database from 2015 onward. The 2015–2025 search window was chosen to give a decade-long evidence base aligned with the Gavi MICs 5.0 period (2021–2025). Extending back to 2015 provides deliberate overlap with the search by Nagi et al. (2021) [12], which ran to December 2019, allowing our review to build on and complement their work while offering scope to capture any studies falling outside their search parameters. Forward and backward citation searches and targeted supplementary searches were also undertaken using Google Scholar. Eligible studies included cost–effectiveness, cost–utility, cost–benefit, cost minimisation, and budget impact analyses, including pre- and post-introduction assessments. The vaccines of focus were RV vaccination in children aged ≤5 years; one- or two-dose HPV vaccination in girls aged 9–14 years and women aged 15–20 years; and PCV in children aged ≤5 years according to WHO guidelines (Appendix A). The geographies of interest were MENA MICs eligible under the Gavi MICs approach (5.0). Country income classifications follow the World Bank’s 2024 definitions (Appendix B) [4,18].

Primary outcomes included incremental cost–effectiveness ratios (i.e., cost per quality-adjusted life year (QALY) or disability-adjusted life year (DALY) gained/averted and cost per case averted), budget impact, cost–benefit metrics (cost–benefit ratio and return on investment), and total vaccination costs. Secondary outcomes included clinical inputs (vaccine efficacy and vaccine coverage assumptions), cost inputs (direct and indirect costs), economic outcomes (healthcare cost savings and productivity gains), and health outcomes (deaths averted, cases averted, and hospitalisations averted).

Articles published in languages other than English were eligible and were screened using translations generated with Google Translate. Search strategies were ran per pathogen in PubMed and by countries of interest in Tufts CEA Registry and the INAHTA database. Full search strategies are presented in the Supplementary Materials (Tables S1–S5).

### 2.3. Study Selection

Title and abstract screening were conducted by two reviewers using the Covidence platform. The reviewers both independently evaluated all studies according to the inclusion and exclusion criteria (Appendix C). Eligible studies were economic evaluations of HPVs, PCVs, or RVs in the relevant target populations conducted in Algeria, Egypt, Iran, Jordan, Lebanon, Morocco, Palestine, and Tunisia; published between 2015 and 2025; and reported outcomes such as incremental cost–effectiveness ratios (ICERs), cost per DALY/QALY averted, and budget impact. Following title/abstract screening, full texts were reviewed to confirm eligibility against the study’s inclusion criteria. Discrepancies were resolved through discussion between the two reviewers, with arbitration by a third independent reviewer when needed.

### 2.4. Data Extraction and Analysis

A data extraction tool was developed in Microsoft Excel, reviewed by a health economist and piloted by two researchers on the same study. Data were extracted into predefined categories (general study characteristics, model characteristics, included costs, main outcomes, and quality appraisal). One reviewer extracted data for all included studies, and a second reviewer independently verified a random 25% sample, focusing on key inputs and outcomes. If substantive discrepancies were identified, verification was expanded to a larger sample (up to all studies). Any uncertainties were resolved through discussion, with adjudication by a third reviewer when required. Given the methodological and clinical heterogeneity across economic evaluations, quantitative synthesis was not undertaken. Instead, findings were synthesised narratively and summarised descriptively. Results were stratified by vaccine type, population group, country income level, and analytic perspective where feasible. Results of individual studies and syntheses were presented in summary tables. Cost–effectiveness ratios (i.e., cost per QALY/DALY gained/averted and cost per case averted) were extracted and reported as presented in the original studies and then expressed as a proportion of gross domestic product (GDP) per capita for the relevant country, as well as the year in which the results were reported. This approach improves interpretability for decision-makers by anchoring cost–effectiveness ratios to a familiar economic benchmark. Country- and year-specific GDP per capita values, expressed in US dollars, were obtained from the World Bank database [19]. Where studies reported cost–effectiveness ratios in local currency, these were converted to US dollars using the World Bank exchange rate database for the corresponding year [20]. To improve comparability, direct medical cost inputs reported across studies were expressed as a percentage of current health expenditure per capita for the relevant country and the year to which the original costs referred. For this purpose, we used the World Bank database [21] on current health expenditure per capita (US dollars). Where studies reported cost inputs in local currency, these were converted to US dollars using the World Bank exchange rate database [20] for the corresponding year. Generative AI was not used in the analysis of included studies.

### 2.5. Quality Assessment of Included Studies

Both the Joanna Briggs Institute (JBI) Critical Appraisal Checklist for Economic Evaluations and the Consolidated Health Economic Evaluation Reporting Standards (CHEERS) 2022 [22] Reporting Guideline were used to assess the quality of the economic studies. The JBI checklist evaluates the risk of bias of included studies. CHEERS is a reporting guideline rather than a formal risk-of-bias assessment tool and was used to assess the transparency and robustness of reporting. CHEERS was included as reporting quality is a relevant dimension of overall study quality in economic evaluations. Overlapping items were removed to avoid double-counting of similar items in the combined quality appraisal tool. This led to 12 items on methodological quality and 17 items on reporting quality.

All included studies were quality appraised by one reviewer, with a random 25% sample of these studies independently appraised by a second reviewer. Clarifications or disagreements were resolved using a third reviewer.

## 3. Results

### 3.1. Literature Screening

**Figure 1:**
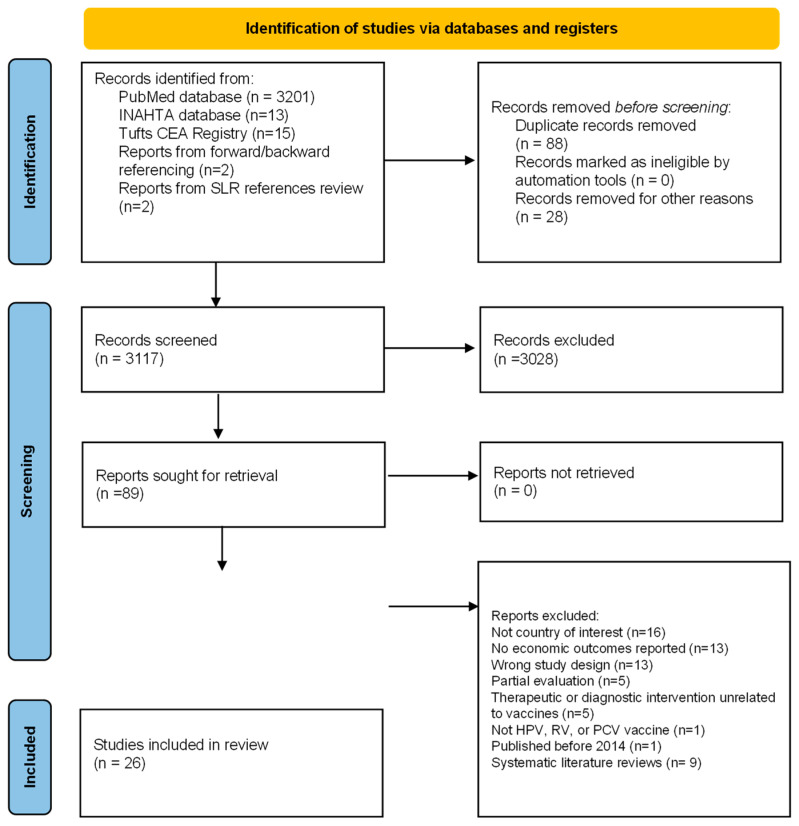
Preferred Reporting Items for Systematic Reviews and Meta-Analyses (PRISMA) flow diagram.

The initial search identified 3233 records. Eighty-eight duplicates and 28 studies were removed for other reasons (15 studies from the Tufts CEA Registry were already identified in the PubMed results and 13 studies from the INAHTA database focussed on other conditions or interventions). Among the 28 studies, 15 studies from the Tufts CEA Registry were already provided by the PubMed database and the 13 studies from the INAHTA database reported irrelevant interventions or conditions. A total of 3117 articles were screened by title and abstract, excluding 3028 records. Eighty-nine records were full-text screened, from which articles were excluded for the following reasons: wrong country setting (*n* = 16); no economic outcomes (*n* = 13); wrong study design (*n* = 13); partial evaluations (*n* = 5); studied therapeutic or diagnostic interventions unrelated to vaccines (*n* = 5); had a vaccine other than those considered (*n* = 1); published before 2015 (*n* = 1); or were systematic literature reviews (*n* = 9), for which only a reference list search was conducted. Partial evaluations, compared to full economic evaluations, include cost-only studies, price surveys, protocols, commentaries, editorials, and reviews. Of the full-text articles assessed, eight studies were considered ambiguous with regards to the eligibility criteria and were excluded following discussion and consensus between reviewers [23,24,25,26,27,28,29,30]. This resulted in a total of 26 studies for final inclusion in the review (Figure 1) [14,15,16,31,32,33,34,35,36,37,38,39,40,41,42,43,44,45,46,47,48,49,50,51,52,53].

### 3.2. Overview of Included Studies

Across 26 included studies, a majority featured HPVs (*n* = 12) [14,16,29,30,31,32,33,34,35,36,37,38] and RVs (*n* = 9) [15,45,46,47,48,49,50,51,52,53], with fewer evaluations of PCVs (*n* = 5) [41,42,43,44,45]. Studies were concentrated in a small number of settings, with country-specific analyses in Iran, Tunisia, Egypt, Morocco, Lebanon, Palestine, and Algeria, as well as several multi-country studies including MENA MICs. Most studies were cost–effectiveness analyses (CEAs) (*n* = 19) [14,15,16,32,33,34,35,36,37,40,41,42,43,45,47,49,50,51,52,53], with a smaller number of cost–benefit analyses (CBAs) [31,39], cost minimisation [33], budget impact [46], and a hybrid of CBA and cost–utility analysis (CUA) designs [44]. Perspectives varied between payer, health system, or governmental, societal, and mixed. Costs were typically reported in USD, with a minority in local currency or international USD. Studies were largely undertaken in pre-introduction contexts (particularly for HPVs), although a small number were conducted after national roll-out of PCVs or rotavirus vaccines in certain countries. We therefore interpret findings primarily as evidence on vaccine policy and financing decisions (including, but not limited to, introduction) rather than assuming all studies specifically evaluate introduction per se. Most studies reported public sector institutional funding (*n* = 13), while fewer studies reported private/industry funding (*n* = 4) or academic funding (*n* = 3), and the remaining studies declared no funding. Key study characteristics and findings are summarised in Table 1. Full data on the characteristics of included studies and findings are available in the Supplementary Materials (Tables S6–S10).

### 3.3. Findings by Vaccine

#### 3.3.1. HPV

Twelve studies focused on HPVs, predominantly in Iran (*n* = 5) [16,32,35,39,40] and Tunisia (*n* = 3) [14,33,36], with additional studies in Morocco (*n* = 1) [37] and Lebanon (*n* = 1) [31], as well as two multi-country analyses that included multiple MENA MICs [34,38]. Most were CEAs [14,16,32,34,35,36,37,40], targeting adolescent girls 9–15 years old [31,32,33,34,35,36,39,40], though a minority assessed broader female age ranges or both sexes [14,16,37]. Most compared vaccination to no vaccination, with some including screening or combination of strategies. Perspectives were mixed, most commonly payer/health system/governmental (*n* = 8) [16,31,32,33,34,35,37,38], with some studies adopting a societal (*n* = 1) [39] or combined governmental–societal perspective (*n* = 2) [36,40].

Most HPV evaluations used static models (*n* = 8), with fewer dynamic modelling approaches (*n* = 3). Time horizons ranged from one year to lifetime when reported, and discounting was commonly applied at 3–5%. For studies evaluating post-introduction, baseline vaccine coverage was not reported in any study. The year of maximum coverage was rarely specified, while assumed final coverage ranged from 70 to 100% when reported.

Most studies included vaccination and direct medical costs. The majority of studies expressed vaccine cost as price per dose, ranging from USD 3.65 (in 2024) for Cecolin^®^ (Xiamen Innovax Biotech Co., Ltd., Xiamen, China) in Tunisia to USD 114 (in 2013) for Gardasil^®^ (Merck & Co., Inc., Kenilworth, NJ, USA) in Iran. Most studies captured direct medical costs for screening, diagnosis and cervical cancer treatment. Cervical cancer treatment costs were consistently higher for later stages. Indirect, direct non-medical (e.g., transportation), and household out-of-pocket costs were included in fewer studies, while delivery, wastage, handling, and injection supply costs were reported only in a subset of studies.

Findings were mixed. Three Iranian studies concluded that HPV vaccination was not cost-effective [32,35,40], although one found vaccinating girls was while vaccinating both sexes less so, with ICERs (USD/QALY) 1.55 and 2.47 times the GDP per capita, respectively [16]. A cost–benefit analysis found that, in Iran, both bivalent and quadrivalent HPV vaccination was economically favourable [39]. In Tunisia, evaluations concluded that HPVs were cost-effective compared to no vaccination (ICERs in USD/DALY between 0.00 and 0.44 times the GDP per capita) [36], while combined vaccination and screening were cost-effective compared to screening alone (ICER in USD/QALY at 0.65 times the GDP per capita) [14]. In the same setting, the HPV vaccination programme with the GAVI price would be the most cost-efficient option (USD 1803 per avoided case), followed by a cervical cancer screening programme with ten-year periodicity (USD 8219 per avoided case), a screening programme with five-year intervals (USD 14,567 per avoided case), a screening programme with three-year intervals (USD 20,479 per avoided case), and a HPV vaccination programme with the manufacturer marketed price (USD 36,854 per avoided case) [33]. In Morocco, vaccination was cost-effective relative to no intervention (ICER in USD/YLS at 0.33 times the GDP per capita) and more efficient than scaling up screening, while vaccination was cost-saving when screening coverage was lower than 15% [37]. A Lebanon-focused evaluation found that HPV vaccination would not be cost-beneficial in 2016, it would cost USD 5,407,790 to vaccinate 38,083 11-year-old girls [31]. Multi-country analyses suggested HPV vaccination is likely to be cost-effective in most countries studied, with ICERs in USD/DALY ranging between 0.47 and 5.37 times the GDP per capita in the MENA MICs [34,38]. Sensitivity analyses were commonly conducted as deterministic (one-way) sensitivity analyses, with only a few studies also reporting probabilistic sensitivity analysis or scenario analyses.

#### 3.3.2. PCV

Five studies evaluated PCV (PCV10 and/or PCV13), conducted in Egypt (*n* = 2) [44,45], Iran (*n* = 1) [41], Tunisia (*n* = 1) [42], and Algeria/Tunisia (*n* = 1) [43]. Most were CEAs [41,42,43,45], and all studies were on young children. Most studies compared PCV10 and/or PCV13 to no vaccination, with one comparing PCV13 to PCV10. Perspectives were primarily payer/governmental (*n* = 3) [42,43,45], with fewer studies adopting a mixed payer–societal perspective (*n* = 2) [41,44].

Most PCV studies used a static model (*n* = 4), with one including a dynamic model. Time horizon ranged from one year to lifetime, and 3% discounting was consistently applied. Baseline vaccine coverage was generally not reported, and only a minority of studies reported the year of maximum coverage. Final coverage assumptions, where reported, ranged from 87% to 100%.

PCV studies consistently included vaccination and direct medical costs. PCV10 and PCV13 price per dose ranged from USD 12.85 in Egypt and Tunisia (in 2016 and 2020, respectively) to USD 20 in Iran (in 2014). Studies primarily presented direct medical costs per episode stratified by syndrome (e.g., AOM, pneumonia, meningitis, NPNM) and care setting (outpatient versus inpatient). Across studies, inpatient episode costs were consistently higher than outpatient costs. Also, costs varied between countries; for example, the ratio relative to current healthcare expenditure per capita of the inpatient cost of meningitis was 5.74 in Algeria and 3.58 in Tunisia in 2016. Only a few studies reported sequelae costs, notably for meningitis. Indirect costs were not included. Delivery and often wastage costs were reported, while handling and injection supply costs were less commonly reported.

Results indicated that PCV13 would be cost-effective compared to no vaccination in Egypt (ICER in USD/QALY was 0.28 [44] and 1.29 [45] times the GDP per capita) and Iran (ICER in USD/DALY was 0.33 and 0.27 times the GDP per capita from a governmental and societal perspective, respectively) [41]. Both PCV13 and PCV10 were cost-effective in Algeria and Tunisia, with ICERs (USD/QALY) ranging between 0.07 and 0.36 times the GDP per capita [43]. The 10-valent pneumococcal non-typeable haemophilus influenzae protein D conjugate vaccine (PHiD-CV) was more cost-effective (ICER in USD/QALY was 0.14 times the GDP per capita) than PCV13 in a Tunisia-focused study [42]. Sensitivity analyses were most commonly deterministic (one-way) across studies, with two studies also using probabilistic sensitivity analysis, one using multivariate, and one using scenario analysis. Serotype coverage was handled differently across studies. The two single-product analyses applied fixed coverage from global estimates (70–74%) [41,45], whereas the three product comparison studies used country-specific serotype distribution to derive efficacy [42,43,44]. Replacement over time was modelled in only two studies, both using dynamics from high-income settings rather than local data [44,45].

#### 3.3.3. RV

Nine studies assessed RVs, including four Iran-specific analyses [46,50,51,53], one State of Palestine study [47], one Morocco study [15], and three multi-country analyses including MENA MICs [47,49,52]. Most were CEAs [15,47,49,50,51,52,53] in children under five [15,47,48,49,50,51,52,53]. Most studies compared RV vaccination to no vaccination, while three compared multiple vaccine products. Perspectives were mixed, most commonly health system/payer perspectives [46,52,53], with several studies also taking a societal perspective [15,47,48,50,51] and one study taking only the societal perspective [49].

RV evaluations used static models. Time horizons ranged from one year to lifetime, and almost all studies applied a 3% discounting. Baseline vaccine coverage was not reported. The year of maximum vaccine coverage was infrequently reported, while assumed final coverage ranged from 90% to 99% when reported.

Almost all RV studies included vaccination and direct medical costs. Most studies expressed vaccine cost as price per dose, ranging from USD 1 (in 2018) to USD 14.77 (in 2018) for Rotarix^®^, RotaTeq^®^, Rotasiil^®^, and Rotavac^®^ in multiple MENA MICs. Studies reporting gastroenteritis costs focused largely on direct medical costs per outpatient visit and per hospitalisation, sometimes distinguishing non-severe and severe episodes. The ratio relative to current healthcare expenditure per capita for direct medical costs ranged from 0.005 (in 2013) per outpatient visit of non-severe rotavirus case to 2.887 (in 2018) for the treatment of sequelae (intussusception) per case in Iran. Across settings, hospitalisation costs were consistently higher than outpatient costs. A subset of studies reported sequelae costs (e.g., intussusception). Half of the studies also included indirect costs. A minority of studies reported direct non-medical costs, such as transportation. Wastage costs were frequently reported, delivery and handling costs were reported in almost half of the studies, while injection supply costs were not reported.

In Iran, RV vaccination was highly cost-effective in several studies, with ICERs (USD/DALY) ranging between 0.00 and 2.05 times the GDP per capita [50,51,53], and was cost-saving from the societal perspective in one study [51]. A budget impact analysis in Iran concluded that the inclusion of rotavirus vaccine in the national vaccination programme would significantly impact health budgets by an incremental cost of USD 131,450,210 (2017) during the 5 years of immunisation [46]. In Morocco, a two-dose human rotavirus, live, attenuated oral vaccine (HRV, Rotarix) schedule was cost-saving compared with three-dose schedules [15]. In the State of Palestine, compared to no vaccination, monovalent RVs were cost-effective from the health system perspective (ICERs in USD/DALY were 0.35 times the GDP per capita for Rotarix, and 0.10 times the GDP per capita for Rotavac) and cost-saving from the societal perspective (ICERs in USD/DALY were −0.22 times the GDP per capita for Rotarix, and −0.48 times the GDP per capita for Rotavac) [47]. Most multi-country evidence was broadly supportive. One analysis across 63 MICs found a high probability that the RV is cost-effective in most countries, with ICERs (USD/DALY) in MENA MICs ranging from cost-saving to 8.00 times the GDP per capita from the government perspective [48]. A second analysis across 137 LMICs concluded that current live oral rotavirus vaccines (LORVs) remain a good investment and that next-generation RVs with comparable or superior efficacy are likely to be cost-effective in most LMICs [49]. Another multi-country study concluded RVs were cost-effective in most analysed countries without reporting country-specific ICERs [52]. Sensitivity analyses were generally deterministic (one-way), with several studies additionally conducting probabilistic sensitivity analysis and a few studies conducting scenario analyses.

### 3.4. Quality Assessment

Methodological and reporting quality varied widely. On the JBI checklist, the maximum possible score is 24. JBI scores ranged from 14 to 24 (average 22, median 23). With the use of appropriate uncertainty analysis and the consideration of generalisability to target setting exhibiting the greatest variability. Some domains, like the identification, measurement and valuation of relevant outcomes, were not applicable in some studies due to design. On the CHEERS checklist, the maximum possible score is 34. CHEERS reporting scores ranged from 21 to 34 (average 30, median 31). Common reporting limitations included insufficiently stating and justifying the time horizon, reporting and justifying the discount rate and discussing heterogeneity. A summary of the quality assessment results is provided in Table 2. The detailed quality assessment results are available in Supplementary Material S6 (Tables S11 and S12).

## 4. Discussion

This systematic review mapped and critically appraised economic evaluations of PCVs, HPVs, and rotavirus vaccines in MENA middle-income countries published between 2015 and 2025. Most studies concluded that vaccination could represent good value for money. However, conclusions were often highly contingent on vaccine price, achievable coverage and assumptions on disease burden, and many evaluations provided limited insight into affordability, budget impact and delivery feasibility. These findings align with prior regional syntheses showing that vaccine economic evidence in MENA is uneven in coverage and variable in quality, with persistent gaps across countries and limited incorporation of key programmatic and indirect effects [12].

With PCV and rotavirus vaccine introduction consistently being cost-effective or cost-saving, delays carry a real public health opportunity cost, as preventable cervical cancer, childhood pneumonia, and rotavirus mortality continue to accrue in countries yet to introduce these vaccines [11]. A purely economic lens also underweights indirect (herd) protection [54], the contribution of PCVs to antimicrobial resistance containment [55], and the gender-equity and cervical cancer elimination dimensions of HPV vaccination [56].

Vaccine price was repeatedly a dominant driver of cost-effectiveness, with some results shifting from borderline to favourable under subsidised or negotiated prices. For example, a Tunisian modelling study noted that, because the country was not Gavi eligible, the cost of HPVs must be met domestically, making price reduction critical to programme continuity [14]. More broadly, many MENA MICs report paying relatively higher prices than comparable countries, partly owing to Gavi ineligibility [57,58]. Practical strategies to improve affordability include improving price visibility through WHO market intelligence and vaccine purchase datasets [59], leveraging pooled or external procurement where feasible, including through UNICEF Supply Division and regional pooled mechanisms [60,61], and using MICs focussed financing modalities such as UNICEF’s Middle Income Countries Financing Facility. This leverages UNICEF procurement scale and can provide prefinancing and contracting options to support access [62].

Yet most evaluations treated vaccine price as fixed, when in practice it is shaped by procurement decisions, product selection, tender design, and volume commitments. Decision relevance would improve if studies routinely tested price–coverage scenarios and reported switch-points—the prices and coverage levels at which cost–effectiveness conclusions change—allowing ministries to align economic results with procurement strategy from the outset rather than treating it as a separate downstream step.

The uneven geography of the evidence presented in this review constrains the transferability of the cost–effectiveness findings. Country-specific evaluations cluster in Iran, Tunisia, Morocco, and Egypt, while the State of Palestine and Lebanon are represented by single studies, with the latter alone concluding HPV vaccination would not be cost-beneficial under prevailing conditions [31,47]. Smaller MICs differ in ways that limit transfer, including small birth cohorts that weaken procurement leverage and raise per-dose delivery costs, macroeconomic and currency volatility (as in Lebanon), and health system fragmentation and displacement (as in the State of Palestine) that affect both costs and achievable coverage. Conclusions drawn predominantly from the larger settings should therefore be treated as hypothesis-generating for the region’s smaller MICs, where locally parameterised analyses are a priority.

Most studies applied thresholds of one to three times GDP per capita, following common practice [12], but this approach has been widely criticised for not reflecting opportunity costs or ability to pay [63]. Favourable ICERs for PCVs and RVs were commonly reported, yet most studies lacked the budgetary and programmatic detail needed to assess real-world affordability. ICER thresholds often translate poorly to budget-constrained MICs, where country-specific thresholds may be far lower, so some favourable conclusions may not hold locally [64]. This review presents ICERs as a proportion of GDP per capita to enable readers to reassess conclusions under alternative thresholds, but GDP normalisation cannot address other sources of heterogeneity, including analytic perspective, costing scope and outcome definitions, so cross-study comparisons should remain cautious. Threshold-based conclusions were frequently presented as binary without quantifying decision uncertainty. Routine reporting of cost–effectiveness acceptability curves (CEACs) and threshold-independent measures, such as net monetary benefit (NMB) or net health benefit (NHB), would improve transparency, particularly where the appropriate threshold is contested or evolving. Economic evaluation is likely more useful for decision making as a coherent evidence package spanning budget impact showing medium-term affordability, fiscal space, and broader value frameworks that capture equity, financial risk protection, and delivery feasibility, with ministries of finance engaged alongside health [65].

Many evaluations were limited in their ability to inform implementation planning. Studies frequently omitted delivery and injection supply costs, wastage, baseline coverage assumptions, and the marginal costs of reaching underserved populations. For ministries, these omissions constrain budgeting and logistics planning, particularly for large birth cohorts. Field reports describe how cold-chain failures, blocked delivery routes, shortages of human resources, and distrust in public health messaging undermine vaccine programmes in Palestine, Yemen, Jordan, and other countries [57]. Models that exclude such programmatic elements may overstate implementable value.

Only a minority of studies used dynamic transmission models capturing herd effects or serotype replacement, despite their relevance where indirect protection materially shapes impact. Such indirect effects are realised only under adequate coverage and delivery, so evaluations built on weak implementation assumptions risk understating achievable benefit. A review of vaccine CEAs in low- and middle-income countries found that only 18% of 243 studies included herd immunity, and only a quarter of these used dynamic models; including herd immunity consistently made ICERs more favourable [66]. Methodological heterogeneity was also substantial, with variation in perspectives, outcomes and threshold choices limiting comparability. Differences in reported ICERs may therefore reflect methodological scope—particularly inclusion of indirect costs—as much as underlying value. Future evaluations would be more decision-useful if they routinely reported results under both payer and societal perspectives, with and without indirect costs.

Funding patterns reinforce the need for transparency. While industry sponsorship does not invalidate findings, it heightens the importance of scrutinising assumptions around price, efficacy or effectiveness and coverage, and underlines the value of independent analyses. The limited proportion of government- or academic-funded work has been noted previously in the region [12].

Several limitations should be acknowledged. Embase, Scopus, and Web of Science were not searched due to lack of institutional access, and grey and unpublished evidence was not sought through dedicated grey literature databases or ministry/agency websites, so some relevant published and unpublished studies may have been missed. This risk was mitigated through searches of economic evaluation and HTA databases, backward and forward citation searching, and targeted Google Scholar searches. Synthesis was also constrained by inconsistent reporting of currency, price year, costing components and implementation assumptions, limiting standardisation. Heterogeneity in models, outcomes and perspectives necessitated a structured narrative synthesis rather than quantitative pooling.

Future economic evaluations in MENA MICs would benefit from a standardised regional reference case and minimum reporting set, specifying perspective, price year, costing scope, and threshold reporting to improve cross-country comparability. Building on the gaps identified above, priorities include locally generated micro-costing of delivery and wastage, budget impact and fiscal space analysis, and dynamic modelling where indirect effects matter. Routine probabilistic analysis, threshold price analysis and distributional equity analysis would further strengthen decision-relevance.

Even a clear economic case changes practice only if there is a pathway to act on it. That pathway usually runs through National Immunization Technical Advisory Groups (NITAGs), yet their functionality is uneven in the region, with only around 62% of Eastern Mediterranean countries meeting all WHO criteria in 2022 [67,68], so MICs facing the sharpest affordability constraints may be least equipped to act. Strengthening NITAG capacity alongside better economic evidence would help ensure that, where vaccination represents good value, it translates into timely introduction.

## 5. Conclusions

In summary, the published evidence generally supports PCV, HPV and rotavirus vaccination as potentially high-value interventions in MENA middle-income countries, but conclusions depend on procurement prices, coverage and burden assumptions, and many studies remain limited for affordability and implementation planning. On this basis, the following policy recommendations are suggested:

- Use pooled procurement and MIC financing—leverage UNICEF Supply Division, regional pooled purchasing, and the UNICEF MIC Financing Facility to counter the higher prices non-Gavi MENA MICs pay.
- Collect local cost data—generate country-specific delivery, wastage, and cold-chain cost data to ground affordability and budget impact estimates.
- Pair CEA with budget impact analysis—require affordability and budget impact evidence, not cost–effectiveness ratios alone, in introduction decisions.

## Figures and Tables

**Table 1:** Summary of included economic evaluations and findings.

Author	Year Published	Study Type	Country	Vaccine	Intervention and Comparator	Perspective	Model Type and Time Horizon	Health Outcome	Main Findings (ICER)	Cost–Effectiveness Threshold	Main Conclusions
	Human Papillomavirus Vaccine (HPV)										
Bahr et al. [31]	2019	CBA	Lebanon	Cervarix™	HPV vaccination campaign vs. no vaccination	Implied: governmental and payer (National Treasury)	Static (no time horizon reported)	No health outcome	USD 5,407,790 to vaccinate 38,083 11-year-old girls	N/A	HPV vaccination would not be cost-beneficial under the circumstances existing in 2016.
Bashari et al. [32]	2025	CEA	Iran	Bivalent, quadrivalent, and nine-valent HPV	HPV vaccination vs. no vaccination	Payer	Static (80 years)	QALYs gained, cervical cancer death reduction, cervical cancer cases reduction	2 doses: Bivalent = USD 33,179/QALY Quadrivalent = USD 45.088/QALY Nine-valent = USD 50.067/QALY 3 doses: Bivalent = USD 50,264/QALY Quadrivalent = USD 69.384/QALY Nine-valent = USD 76.778/QALY	1× GDP per capita as threshold, based on World Bank data	None of the vaccination strategies were considered cost-effective.
Gamaoun, R. [33]	2018	CMA	Tunisia	Bivalent HPV	National HPV vaccination programme vs. cervical cancer screening using the Pap smear test	Implied: health system	Static (1 year)	Cervical cancer cases avoided	Ascending incremental costs by avoided cervical cancer case: 1—the national vaccination programme through GAVI support (USD 1803); 2—the cervical cancer screening according to 10-year periodicity (USD 8219); 3—the cervical cancer screening according to 5-year periodicity (USD 14,567); 4—the cervical cancer screening according to 3-year periodicity (USD 20,479); and 5—the national vaccination programme according to the manufacturer marketed price (USD 36,854)	N/A	HPV national vaccination programme combined with cervical cancer screening according to 5-year periodicity present the best cost-effective strategy for cervical cancer prevention.
Hagens et al. [16]	2024	CEA	Iran	HPV	HPV vaccination vs. no vaccination	Healthcare system	Dynamic (70 years)	QALYs gained, deaths averted	Vaccinating boys and girls: USD 7916/QALY Vaccinating only girls: USD 4949/QALY Vaccinating only boys: USD 15,529/QALY	ICER below 1× GDP per capita	Vaccinating only girls was found to be cost-effective, with an ICER close to the GDP per capita. Vaccinating both sexes was shown to be less cost-effective compared to girls only, and vaccinating boys only was not found to be cost-effective, with an ICER between one and three times, and greater than three times the GDP per capita.
Jit et al. [34]	2014	CEA	179 countries, including Algeria, Egypt, Iran, Jordan, Lebanon, Morocco, and Tunisia	HPV 16/18	HPV16/18 versus no further vaccine introductions; HPV16/18 versus increased vaccine introductions in 2012–2032	Implied: healthcare or purchaser	Static (1 year)	Deaths prevented, cancers prevented, DALYs averted	Cost per life year saved: Algeria: USD 4250 Egypt: USD 13,900 Iran: USD 10,800 Jordan: USD 11,700 Lebanon: USD 6760 Morocco: USD 1700 Tunisia: USD 6500 Cost per DALY prevented: Algeria: USD 3830 Egypt: USD 12,600 Iran: USD 9940 Jordan: USD 10,800 Lebanon: USD 6180 Morocco: USD 1560 Tunisia: USD 5970	GDP per capita	HPV vaccination is likely to be very cost-effective in most countries and cost-effective in almost every country in the world.
Khatibi et al. [35]	2014	CEA	Iran	HPV 16/18 (Gardasil^®^)	HPV vaccination vs. no vaccination	Governmental	Static (59 years)	QALYs gained	IRR 439,092,468/QALY	ICER below 3× GDP per capita	Quadrivalent HPV (Gardasil^®^) is not cost-effective in Iran based on the base case parameters value.
Khiari et al. [14]	2024	CEA	Tunisia	HPV 16/18	HPV 16/18 vaccination vs. no vaccination; combined HPV vaccination and screening vs. screening alone	Not reported	Dynamic (no time horizon reported)	QALYs gained	Combined vaccine and screening strategy vs. no intervention: USD 966.5/QALY Combined vaccine and screening strategy vs. screening alone: USD 2293.3/QALY	ICER less than the country’s GDP per capita	Compared with screening alone, the implementation of HPV vaccination in addition to the current cytology screening programme in Tunisia would be considered cost-effective on the basis of the threshold GDP per capita.
Laraj et al. [13]	2025	CEA	Tunisia	Cecolin^®^, Cervarix™, Gardasil-4™, Gardasil-9^®^	HPV vaccination vs. no vaccination	Governmental and societal	Static (lifetime)	DALYs averted, deaths averted, cases averted	Cecolin^®^ PRIME model: Government perspective: USD 162/DALY Societal perspective: USD 34/DALY UNIVAC model: Government perspective: USD 121/DALY Societal perspective: USD 13/DALY Cervarix™ PRIME model: Government perspective: USD 845/DALY Societal perspective: USD 718/DALY UNIVAC model: Government perspective: USD 625/DALY Societal perspective: USD 516/DALY Gardasil-4™ PRIME model: Government perspective: USD 1266/DALY Societal perspective: USD 1139/DALY UNIVAC model: Government perspective: USD 935/DALY Societal perspective: USD 827/DALY Gardasil-9^®^ PRIME model: Government perspective: USD 1845/DALY Societal perspective: USD 1718/DALY UNIVAC model: Government perspective: USD 1362/DALY Societal perspective: USD 1253/DALY Cecolin^®^ (cross-protection) PRIME model: Government perspective: USD 151/DALY Societal perspective: USD 24/DALY UNIVAC model: Government perspective: USD 113/DALY Societal perspective: USD 5/DALY Cervarix™ (cross-protection) PRIME model: Government perspective: USD 686/DALY Societal perspective: USD 559/DALY UNIVAC model: Government perspective: USD 507/DALY Societal perspective: USD 399/DALY	A range of alternative possible WTP thresholds up to 0.3 times the national GDP per capita	The four HPVs (Cecolin^®^, Cervarix^®^, Gardasil-4^®^, and Gardasil-9^®^,) were cost-effective in the Tunisian context.
Messoudi et al. [37]	2019	CEA	Morocco	HPV 16/18	Three strategies modelled: (1) Screening women (30–49 years old) versus no intervention (2) HPV16/18 vaccination of girls at least 14 years old versus no intervention (3) Combined vaccination and screening versus screening alone	Healthcare system	Static (lifetime)	Years of life saved (YLS)	Strategy: Visual inspection with acetic acid 5% ICER for vaccinating 14-year-old girls: dominated ICER for vaccination + screening: USD 2327/YLS Strategy: Visual inspection with acetic acid 15% ICER for vaccinating 14-year-old girls: dominated ICER for vaccination + screening: USD 2911/YLS Strategy: Visual inspection with acetic acid 20% ICER for vaccinating 14-year-old girls: USD 1150/YLS ICER for vaccination + screening: USD 3743/YLS Strategy: Visual inspection with acetic acid 30% ICER for vaccinating 14-year-old girls: USD 1150/YLS ICER for vaccination + screening: USD 5308/YLS Strategy: Visual inspection with acetic acid 50% ICER for vaccinating 14-year-old girls: USD 1150/YLS ICER for vaccination + screening: USD 10,712/YLS Strategy: Visual inspection with acetic acid 70% ICER for vaccinating 14-year-old girls: USD 1150/YLS ICER for vaccination + screening: USD 14,170/YLS Strategy: Visual inspection with acetic acid 100% ICER for vaccinating 14-year-old girls: USD 1150/YLS ICER for vaccination + screening: USD 20,167/YLS	ICER below 3× GDP per capita	HPV vaccination could be highly effective and cost-effective in Morocco. Current screening would be good value for money compared with no intervention, but scaling up screening coverage would make it inefficient compared with vaccination.
Rosettie et al. [38]	2021	Cost–effectiveness and meta-regression analyses	195 countries, including Iran and Egypt	HPV quadrivalent and bivalent vaccines	HPV vaccination vs. no vaccination	Healthcare payer	Static (lifetime)	DALYs averted	Algeria: USD 6369 (1169 to 20,202)/DALY averted Egypt: USD 10,057 (1854 to 31,337)/DALY averted Iran: USD 9222 (1683 to 28,936)/DALY averted Jordan: USD 10,438 (1921 to 32,502)/DALY averted Lebanon: USD 4196 (1246 to 15,793)/DALY averted Morocco: USD 5317 (979 to 16,942)/DALY averted Palestine: USD 9632 (1773 to 30,034)/DALY averted	0.5, 1, and 3× GDP per capita	The authors concluded that their results identified countries where the HPV had good value, but whether the vaccine is cost-effective based on country-specific predicted ICERs and cost–effectiveness thresholds is not reported.
Sargazi et al. [39]	2022	CBA	Iran	HPV bivalent and quadrivalent vaccine	HPV vaccination vs. no vaccination	Societal	Not reported (lifetime)	Net benefit	Cost–benefit ratio (willingness to pay approach): Bivalent: −USD 15.11 Quadrivalent: USD 2.51 Cost–benefit ratio (cost of illness approach): Bivalent: USD 258.12 Quadrivalent: USD 43.51	N/A	This study confirmed the benefits of the national bivalent and quadrivalent vaccination programmes. Specifically, the bivalent vaccine’s benefit is higher than its cost under the cost-of-illness method; the quadrivalent vaccine’s benefit is higher than its cost under both evaluation methods (cost-of-illness and willingness to pay).
Yaghoubi et al. [40]	2018	CEA	Iran	HPV 16/18 (Gardasil^®^)	HPV vaccination vs. no vaccination	Governmental and societal	Dynamic (10 years)	DALYs averted, cases averted, deaths averted	Governmental: USD 15,205/DALY Societal: USD 14,999/DALY	Threshold using GDP per capita, using the WHO definition	Introducing a three-dose HPV vaccination programme is currently not cost-effective in Iran.
Pneumococcal Vaccine (PCV)											
Ezoji et al. [41]	2019	CEA	Iran	PCV13	PCV vaccination vs. no vaccination	Governmental and societal	Static (10 years)	DALYs averted, cases averted, deaths averted, hospital admissions averted, outpatient visits averted	Governmental perspective: USD 1890/DALY Societal perspective: USD 1538/DALY	1 and 3× GDP per capita (2012)	Introduction of PCV-13 for children under 5 years in the Islamic Republic of Iran would be cost-effective.
Lagoubi et al. [42]	2022	CEA	Tunisia	PCV13 or PhiD-CV	PCV vaccination vs. no vaccination	Payer	Dynamic (lifetime)	QALYs gained, deaths averted, cases averted, medical visits averted, hospitalisations averted	PCV13: dominated PhiD-CV: USD 484/QALY	GDP per capita	PCVs are a cost-effective strategy to relieve the burden associated with diseases caused by S. pneumoniae and NTHi in Tunisia. PHiD-CV is more cost-effective than PCV13, generating similar health benefits at a reduced net cost of almost $1 million USD per vaccinated cohort.
Pugh et al. [43]	2018	CEA	Algeria, Tunisia	PCV10, PCV13	PCV13 or PCV10 vs. no vaccination	Payer	Static (1 year)	QALYs gained, deaths averted, cases averted	PCV13 in Tunisia: USD 848/QALY PCV10 in Tunisia: USD 1366/QALY PCV13 in Algeria: USD 308/QALY PCV10 in Algeria: USD 731/QALY	If the vaccines averted 1 QALY less than 3× GDP per capita, they are cost-effective. They are highly cost-effective if they are below 1× GDP per capita	PCV NIPs are highly cost-effective and highly impactful public health interventions.
Sevilla et a [44]	2022	CUA and CBA	Egypt	PCV10 (Synflorix™), PCV13 (Prevenar13^®^)	PCV13 or PCV10 vs. no vaccination; PCV13 vs. PCV10	Societal (CBA) and payer (CUA)	Static (100 years)	QALYs gained, deaths averted, cases averted, hospitalisations averted	PCV13 vs. no programme: USD 925.6 (512.08–1734.9)/QALY PCV10 vs. no programme: USD 1984.41 (1186.32–3804.67)/QALY PCV13 vs. PCV10: USD 173.98 (87.59–331.23)/QALY	1 to 3 × GDP per capita	A universal paediatric PCV13 programme represents good value for money for policymakers in Egypt.
Sibak et al. [45]	2015	CEA	Egypt	PCV13	PCV vaccination vs. no vaccination	Governmental	Static (10 years)	DALYs averted, deaths averted, cases averted, inpatient admissions averted, outpatient visits averted	USD 3916/DALY	1× GDP per capita for highly cost-effective 3× GDP per capita for cost-effective	PCVs would be cost-effective from a governmental perspective.
Rotavirus Vaccine (RV)											
Azad et al. [46]	2019	BIA	Iran	RotaTeq^®^ (RV5)	RotaTeq^®^ vs. no immunisation	Health system	Static (5 years)	Outpatient cases averted, inpatient cases averted	Incremental cost during 5 years of immunisation: USD 131,450,210	Not applicable	The inclusion of rotavirus vaccine in the national vaccination programme would have a significant effect on health budgets and would raise government expenditure.
Debellut et al. [47]	2020	CEA	Palestine	Rotarix^®^, Rotavac^®^	Rotarix^®^ vs. no vaccination; Rotavac^®^ vs. no vaccination; Rotavac^®^ vs. Rotarix	Health system and societal	Static (10 years)	DALYs averted, cases averted, deaths averted, outpatient visits averted, hospitalisations averted	Rotarix^®^ compared to no vaccine (health system perspective): USD 1254/DALY Rotarix^®^ compared to no vaccine (societal perspective): Cost-saving (−USD 794/DALY) Rotavac^®^ compared to no vaccine (health system perspective): USD 353/DALY Rotavac^®^ compared to no vaccine (societal perspective): Cost-saving (−USD 1695/DALY) Rotavac^®^ compared to Rotarix^®^ (health system perspective): Cost-saving (−USD 901/DALY) Rotavac^®^ compared to Rotarix^®^ (societal perspective): Cost-saving (−USD 901/DALY)	1× GDP per capita	From the health system perspective, rotavirus vaccination with either Rotarix^®^ or Rotavac^®^ is a cost-effective intervention in Palestine compared to no vaccination. When accounting for averted healthcare-related costs for households, using either vaccine is a cost-saving intervention. When evaluating the switch, Rotavac^®^ presents an economic advantage over Rotarix^®^ and shifting from Rotarix^®^ to Rotavac^®^ was a cost-saving option from both health system and societal perspectives.
Debellut et al. [48]	2021	CEA and benefit-risk analysis	63 MICs, including Algeria, Egypt, Iran, Jordan, Lebanon, Morocco, Palestine, and Tunisia	Rotarix^®^, Rotavac^®^, Rotasiil^®^, and next-generation rotavirus vaccines (NGRVs)	Current rotavirus vaccines vs. no vaccination; NGRVs vs. current rotavirus vaccines	Governmental and societal	Static (10 years)	DALYs averted, cases averted, deaths averted, outpatient visits averted, hospitalisations averted	Governmental perspective: Rotarix^®^: Algeria: USD 8332 (3520–15,992)/DALY Egypt: USD 2936 (2081–3872)/DALY Iran: USD 6669 (3839–9417)/DALY Jordan: USD 9497 (5799–14,016)/DALY Lebanon: USD 6374 (2052–10,352)/DALY Morocco: USD 3502 (2271–4970)/DALY Palestine: USD 10,171 (5609–16,294)/DALY Tunisia: USD 11,335 (6271–17,834)/DALY Rotavac^®^: Algeria: USD 2781 (1024–5418)/DALY Egypt: USD 1048 (657–1370)/DALY Iran: USD 1625 (0–2767)/DALY Jordan: USD 3318 (1671–5003)/DALY Lebanon: CS (CS–1690)/DALY Morocco: USD 1140 (643–1637)/DALY Palestine: USD 3604 (1803–5835)/DALY Tunisia: 3324 (1298–5587)/DALY Rotasiil^®^: Algeria: USD 2278 (789–4530)/DALY Egypt: USD 871 (511–1160)/DALY Iran: USD 1198 (0–2257)/DALY Jordan: USD 2701 (1223–4195)/DALY Lebanon: CS (CS–1029)/DALY Morocco: USD 937 (491–1361)/DALY Palestine: USD 2986 (1430–4927)/DALY Tunisia: USD 2636 (841–4626)/DALY Societal perspective: Rotarix^®^: Algeria: USD 8071/DALY Egypt: USD 2869/DALY Iran: USD 6172/DALY Jordan: USD 9106/DALY Lebanon: USD 4905/DALY Morocco: USD 3410/DALY Palestine: USD 9893/DALY Tunisia: USD 10,842/DALY Rotavac^®^: Algeria: USD 2520/DALY Egypt: USD 982/DALY Iran: USD 1128/DALY Jordan: USD 2927/DALY Lebanon: Cost-saving Morocco: USD 1048/DALY Palestine: USD 3326/DALY Tunisia: USD 2831/DALY Rotasiil^®^: Algeria: USD 2017/DALY Egypt: USD 805/DALY Iran: USD 701/DALY Jordan: USD 2310/DALY Lebanon: Cost-saving Morocco: USD 845/DALY Palestine: USD 2708/DALY Tunisia: USD 2143/DALY	0.5× GDP per capita of each country	In most MICs not eligible for Gavi funding, rotavirus vaccination has a high probability to be cost-effective with a favourable benefit–risk profile.
Debellut et al. [49]	2022	CEA	137 LMICs, including Iran, Egypt, Algeria, Tunisia, Jordan, Lebanon, Morocco, and Palestine	Licenced vaccines: Rotavac^®^, Rotasiil^®^, Rotarix^®^ Other vaccines: RV3-BB, trivalent P2-VP8, trivalent P2-VP8 comprising part of a DTP-containing combination vaccine	Vaccines were compared to no vaccination and to each other	Societal	Static (10 years)	DALYs averted, cases averted, deaths averted, outpatient visits averted, hospitalisations averted	Rotavac^®^ Algeria: USD 2662/DALY Egypt: USD 1093/DALY Iran: USD 1400/DALY Lebanon: Cost-saving Morocco: USD 1271/DALY Palestine: USD 3385/DALY Tunisia: USD 3286/DALY Jordan: USD 3138/DALY Rotasiil^®^ Algeria: USD 3011/DALY Egypt: USD 1221/DALY Iran: USD 1725/DALY Lebanon: Cost-saving Morocco: USD 1429/DALY Palestine: USD 3795/DALY Tunisia: USD 3790/DALY Jordan: 3566/DALY Rotarix^®^ Algeria: USD 12,148/DALY Egypt: USD 4507/DALY Iran: USD 10,063/DALY Lebanon: USD 8802/DALY Morocco: USD 5498/DALY Palestine: USD 14,395/DALY Tunisia: USD 16,788/DALY Jordan: USD 14,647/DALY	0.25 to 1× GDP per capita for each country	The results show that while currently available live oral rotavirus vaccines (LORVs) remain a good investment for countries and donors today, an injectable next-generation rotavirus vaccine (iNGRV) with comparable or superior efficacy to LORVs is likely to be cost-effective in the majority of LMICs.
Javanbakht et al. [50]	2015	CEA	Iran	RotaTeq^®^ (RV5)	Rotavirus vaccination vs. no vaccination	Health system and societal	Static (10 years)	DALYs averted, cases averted, deaths averted, outpatient visits averted, inpatient admissions averted	Governmental perspective: USD 2868/DALY Society perspective: USD 382/DALY	1× GDP per capita (2013) for highly cost-effective 3× GDP per capita (2013) for cost-effective	Introduction of rotavirus vaccine is a highly cost-effective strategy from the government perspective.
Mohy et al. [15]	2024	CEA	Morocco	HRV, HBRV, BRV-PV	Rotavirus vaccination vs. no vaccination	Country payer and societal	Static (no time horizon reported)	QALYs gained	Base case ICER (HRV as the reference) country payer perspective HBRV: HRV is dominant BRV-PV: USD 328,376 Base case ICER (HRV as the reference) societal perspective HBRV: HRV is dominant BRV-PV: HRV is dominant	USD 3500 per QALY	HRV was associated with lower costs versus HBRV from both country payer (−$1.8 M) and societal (−$4.1 M) perspectives, and versus BRV-PV 1-dose vial from the societal perspective (−$187,000), dominating those options in the cost–effectiveness analysis. However, costs of BRV-PV 1-dose vials were lower than HRVs from the payer perspective, resulting in an ICER of approximately $328,376 per QALY, above the assumed cost–effectiveness threshold of $3500. Vaccination with a 2-dose schedule of HRV may be a cost-saving option and could lead to better health outcomes for children in Morocco versus 3-dose schedule rotavirus vaccines.
Mousavi Jarrahi et al. [51]	2015	CEA	Iran	Rotarix^®^	Rotavirus vaccination vs. no vaccination	Healthcare and societal	Static (5 years)	DALYs averted, deaths averted, outpatient visits averted, hospitalisations averted	A cost of 19 USD for each DALY averted from the healthcare system perspective A saving of 278 USD for each DALY averted from the societal perspective	1× GDP per capita	Introducing rotavirus vaccine into EPI programme would be highly cost-effective public health intervention in Iran.
Paternina-Caicedo et al. [52]	2015	CEA	LMICs, including Algeria, Egypt, Jordan, Morocco, and Tunisia	RV1, RV5	RV1 or RV5 vaccination vs. no vaccination	Healthcare	Static (1 year)	DALYs averted, deaths averted	Not reported by country	Below 1× GDP per capita and between 1× and 3× GDP per capita	Rotavirus vaccine is cost-effective in most analysed countries.
Shakerian et al. [53]	2015	CEA	Iran	RotaTeq^®^, Rotarix^®^	RotaTeq^®^ vs. no vaccination; Rotarix^®^ vs. no vaccination	Healthcare	Static	DALYs averted, cases averted	RotaTeq^®^: 3672 episodes per 100,000 with USD 10 price in base year = USD 16,186/DALY 6243 episodes per 100,000 with SD10 price in base year = USD 15,376/DALY 36,000 episodes per 100,000 with USD 10 price in base year = USD 9582/DALY 108,000 episodes per 100,000 with USD 10 price in base year = USD 3701/DALY 3672 episodes per 100,000 with USD 15 price in base year = USD 23,380/DALY 6243 episodes per 100,000 with USD 15 price in base year = USD 22,278/DALY 36,000 episodes per 100,000 with USD 15 price in base year = USD 14,443/DALY 108,000 episodes per 100,000 with USD 15 price in base year = USD 6444/DALY Rotarix^®^: 3672 episodes per 100,000 with USD 10 price in base year = USD 9402/DALY 6243 episodes per 100,000 with USD 10 price in base year = USD 8868/DALY 36,000 episodes per 100,000 with USD 10 price in base year = USD 5000/DALY 108,000 episodes per 100,000 with USD 10 price in base year = USD 1115/DALY 3672 episodes per 100,000 with USD 15 price in base year = USD 13,599/DALY 6243 episodes per 100,000 with USD 15 price in base year = USD 12,895/DALY 36,000 episodes per 100,000 with USD 15 price in base year = USD 7835/DALY 108,000 episodes per 100,000 with USD 15 price in base year = USD 2715/DALY	WHO threshold rate (specific threshold rates were not mentioned)	Assuming that the illness episodes are 100% and 300% for Rotarix^®^ and 300% for RotaTeq^®^, the ratio of cost per DALY averted is highly cost-effective.

CBA: cost–benefit analysis; CEA: cost–effectiveness analysis; CMA: cost minimisation analysis; CUA: cost–utilisation analysis; BIA: budget impact analysis; N/A: not applicable.

**Table 2:** Overall quality assessment scores.

Study	JBI	CHEERS
Bahr et al. [31]	14	21
Bashari et al. [32]	24	29
Gamaoun, R [33]	21	24
Hagens et al. [16]	20	32
Jit et al. [34]	24	32
Khatibi et al. [35]	23	32
Khiari et al. [14]	23	26
Laraj et al. [36]	24	34
Messoudi et al. [37]	23	32
Rosettie et al. [38]	22	30
Sargazi et al. [39]	17	26
Yaghoubi et al. [40]	23	32
Ezoji et al. [41]	22	31
Lagoubi et al. [42]	24	31
Pugh et al. [43]	23	31
Sevilla et al. [44]	24	34
Sibak et al. [45]	23	31
Azad et al. [46]	23	30
Debellut, 2020 [47]	23	30
Debellut, 2021 [48]	24	32
Debellut, 2022 [49]	24	29
Javanbakht et al. [50]	23	32
Mohy et al. [15]	24	30
Mousavi Jarrahi et al. [51]	23	30
Paternina-Caicerdo et al. [52]	24	32
Shakerian et al. [53]	20	29

## Data Availability

Data are available upon reasonable request to the author.

## Supplementary Materials

The following supporting information can be downloaded at: https://www.mdpi.com/article/10.3390/vaccines14070591/s1, Table S1: PubMed search strategy for HPVs; Table S2: PubMed search strategy for PCVs; Table S3: PubMed search strategy for RVs; Table S4: Tufts CEA Registry search strategy and results; Table S5: International HTA database search strategy and results; Table S6: General characteristics of included studies; Table S7: Included costs; Table S8: Vaccine costs included; Table S9: Main outcomes from included studies; Table S10: ICERs used in the study; Table S11: Full JBI item-by-item scoring; Table S12: Full CHEERS item-by-item scoring.

## Abbreviations

The following abbreviations are used in this manuscript:

MENA	Middle East and North Africa
MICs	Middle-Income Countries
HPV	Human Papillomavirus Vaccine
PCV	Pneumococcal Conjugate Vaccine
RV	Rotavirus Vaccine
LMICs	Low- and Middle-Income Countries
CEA	Cost–Effectiveness Analysis
INAHTA	International Health Technology Assessment
QALY	Quality-Adjusted Life Year
DALY	Disability-Adjusted Life Year
GDP	Gross Domestic Product
JBI	Joanna Briggs Institute
CHEERS	Consolidated Health Economic Evaluation Reporting Standards
PRISMA	Preferred Reporting Items for Systematic Reviews and Meta-Analyses
CBA	Cost–Benefit Analysis
CUA	Cost–Utility Analysis
ICER	Incremental Cost–Effectiveness Ratio
PhiD-CV	10-Valent Pneumococcal Non-Typeable Haemophilus Influenzae Protein D Conjugate Vaccine
USD	United States Dollar

## Appendix A

In this section, the vaccine dosing schedules are briefly described according to the latest available WHO 2025 guidelines [69].

### Appendix A.1. New Vaccine Dosing Schedules for PCV (WHO 2025 Guidelines)

PCV:

- Schedule—the World Health Organization (WHO) recommends a three-dose schedule for PCVs. This can be given as either:
  ⚬ Two primary doses + one booster (2p + 1).
  ⚬ Three primary doses with no booster (3p + 0).
  ⚬ Starting age—vaccination can start as early as 6 weeks of age.
  ⚬ Available vaccines—there are three WHO prequalified PCVs available, targeting either 10 or 13 common serotypes. The manufacturers and details are listed in Table A1.

**Table A1:** WHO prequalified PCVs.

Manufacturer	Vaccine	PQ Year	Presentation	Dose/Course
GSK (London, UK)	PCV10	2017	4-dose vial	3
Serum Institute of India (SII) (Pune, India)	PCV10	2019	1-dose vial	3
	PCV10	2019	5-dose vial	3
Pfizer (New York, NY, USA)	PCV13	2010	1-dose vial	3
	PCV13	2016	4-dose vial	3

### Appendix A.2. New Vaccine Dosing Schedules for HPV Vaccine (WHO 2025 Guidelines)

HPV vaccine:

- As of 2025, WHO recommends:
  ⚬ A one- or two-dose schedule for girls aged 9–14 years and women aged 15–20 years.
  ⚬ Two doses with a 6-month interval for women older than 21 years.
- There are four available WHO prequalified HPVs (Table A2).

**Table A2:** WHO prequalified HPV.

Manufacturer	Vaccine	PQ Year	Presentation	Dose/Course
GSK	Bivalent	2009	2-dose vial	1 or 2 dose
Merck & Co./MSD	Quadrivalent	2009	1-dose vial	1 or 2 dose
	Nonavalent	2018	1-dose vial	1 or 2 dose
Xiamen Innovax	Bivalent	2021	1-dose vial	1 or 2 dose

### Appendix A.3. New Vaccine Dosing Schedules for RV Vaccine (WHO 2025 Guidelines)

RV vaccine:

WHO recommends that the first dose of RV vaccine be administered as soon as possible after 6 weeks of age, along with DTP vaccination.

WHO prequalified RV vaccines are:

- Rotarix (GSK): Monovalent, single dose presentation
- RotaTeq (Merck & Co./MSD): Pentavalent, single dose and two-dose presentation
- Rotavac (Bharat Biotech (Telangana, India)): Monovalent, five-dose or 10-dose presentation
- RotaSiil (Serum Institute of India): Pentavalent, single dose and two-dose presentation

## Appendix B

### Definition and Description of MENA Middle-Income Countries

The World Bank states, for the 2024 fiscal year, lower middle-income economies are defined as those with a GNI per capita, calculated using the World Bank Atlas method, of between $1136 and $4495. Country eligibility for the Gavi MICs approach, as of July 2025, includes the following LMICs which will be considered eligible for study inclusion:

- Egypt.
- Jordan.
- Lebanon.
- Morocco.
- Palestine.
- Tunisia.

The following recently transitioned upper middle-income countries will also be included in the review due to Gavi MICs support 5.0 being retained until the end of 2025:

- Algeria.
- Iran (Islamic Republic of).

## Appendix C

### Appendix C.1. PICOTS Framework

Studies were assessed with the PICOT framework as a guide to identify studies for inclusion or exclusion by title and abstract screening, as well as by full-text screening.

**Table A3:** PICOTS criteria for study inclusion.

PICOTS	Characteristic/s
Population(s)	Children ≤ 5 years of age for pneumococcal and rotavirus vaccines. Girls 9–14 years, women aged 15–20 years for HPVs.
Interventions	Human papillomavirus vaccines. Pneumococcal vaccines. Rotavirus vaccines.
Comparator	No vaccination
Outcomes	Primary outcomes: Incremental cost–effectiveness ratios (ICERs), cost per DALY/QALY averted, cost per case averted, budget impact, return on investment, fiscal space implications, total vaccination costs. Secondary outcomes: Parameter values used in models (e.g., vaccine efficacy, coverage, costs, disability weights), healthcare cost savings, productivity gains, deaths averted, cases averted, hospitalisations averted. Herd immunity effect: Indirect protection of unvaccinated persons, whereby an increase in the prevalence of immunity caused by the vaccine prevents the virus propagation in vulnerable populations. Hospitalisation rates of children with rotavirus or pneumonia infections, women with cervical cancer. Incidence/prevalence of rotavirus/pneumonia/HPV infection and cervical cancer (home/community, primary care, hospital).
Timing	Studies published in the last 10 years (2014–2024)
Setting	Middle-income countries in the MENA region, namely Algeria, Egypt, Iran, Jordan, Lebanon, Morocco, Palestine, and Tunisia

### Appendix C.2. Inclusion and Exclusion Criteria

The inclusion and exclusion criteria presented below were used in full-text screening of studies.

**Table A4:** Inclusion and exclusion criteria.

Inclusion Criteria	Exclusion Criteria
Study focuses on human populations relevant to new vaccine introduction (e.g., children, adolescents, adults targeted by NIPs).	Animal studies, hypothetical cohorts unrelated to immunisation policy, or populations outside human vaccine context.
Examines introduction or use of a specific vaccine (HPV, PCV, rotavirus) or compares vaccination with no vaccination/alternative strategy.	Hypothetical cohort unrelated to policy.
Reports economic outcomes such as cost–effectiveness, cost–utility, cost–benefit, budget impact, or ROI.	Studies not focused on HPVs, RVs, or PCVs, as well as therapeutic or diagnostic interventions unrelated to vaccines.
Full economic evaluations (CEA, CUA, CBA, BIA, ROI) using empirical data or model-based analyses.	No economic outcomes reported (e.g., epidemiological or coverage studies only).
Study conducted in or providing data pplicable to MENA countries of interest (Algeria, Egypt, Morocco, Lebanon, Iran, Tunisia, Jordan, and Palestine).	Partial evaluations (cost-only studies, price surveys), commentaries, protocols, editorials, or reviews.
Published from 2014 onward.	Not conducted in a country of interest.
Published in English, French, or Arabic (if translation is feasible).	Published before 2014.
	Not published in English, French, or Arabic.
