# Supplementary material for: Economic Evaluations of New Vaccine Introduction in Middle-Income Countries in the Middle East and North Africa Region: A Systematic Review
Source: Vaccines (Basel). 2026 Jul 2;14(7):591. doi: 10.3390/vaccines14070591 (PMC13431315; doi:10.3390/vaccines14070591)
Supplement: Supplementary file 1 [file vaccines-14-00591-s001.zip › vaccines-4357030-supplementary.pdf]

Supplementary Materials

Supplementary S1: Search Strategy

The following search strategies were utilised. Searches on PubMed were conducted with each disease of interest, namely, human papillomavirus (HPV), pneumococcal virus (PCV) , and rotavirus (RV).

Table S1. PubMed search strategy for HPVs

Search number	Query	Results
1	"Human Papillomavirus Viruses"[Mesh]	9,207
2	"Alphapapillomavirus"[Mesh]	10,977
3	"Papillomavirus Infections"[Mesh:NoExp]	38,074
4	"Uterine Cervical Neoplasms"[Mesh]	91,800
5	Human papilloma virus[Title/Abstract]	8,431
6	Human papillomavirus[Title/Abstract]	49,483
7	Papillomavirus infection[Title/Abstract]	4,534
8	HPV infection[Title/Abstract]	15,165
9	Cervical neoplasm[Title/Abstract]	308
10	Cervical cancer[Title/Abstract]	69,609
12	"Immunization"[Mesh]	227,061
13	"Immunotherapy, Active"[Mesh:NoExp]	2,523
14	"Immunization Programs"[Mesh:NoExp]	13,935
15	"Vaccination"[Mesh]	121,695
16	("Vaccination"[Mesh]) OR "Mass Vaccination"[Mesh]	121,695
17	vaccine[Title/Abstract] OR vaccination[Title/Abstract] OR immunisation[Title/Abstract] OR immunization[Title/Abstract]	456,892
18	#1 OR #2 OR #3 OR #4 OR #5 OR #6 OR #7 OR #8 OR #9 OR #10	154,382
19	#12 OR #13 OR #14 OR #15 OR #16 OR #17	538,380
20	#18 AND #19	18,656
21	"Papillomavirus Vaccines"[Mesh]	11,665
22	"Human Papillomavirus Recombinant Vaccine Quadrivalent, Types 6, 11, 16, 18"[Mesh]	844
23	(Papillomavirus Vaccine[Title/Abstract] OR HPV Vaccine[Title/Abstract] OR Human Papillomavirus Recombinant Vaccine[Title/Abstract])	8,403
24	Gardasil[Title/Abstract]	688
25	HPV L1 Vaccine[Title/Abstract]	4
26	Cecolin[Title/Abstract]	24
27	#21 OR #22 OR #23 OR #24 OR #25 OR #26	14,043
28	#20 OR #27	20,217
29	"Economics"[Mesh:NoExp]	27,552
30	"Costs and Cost Analysis"[Mesh]	282,230
31	"Economics, Dental"[Mesh:NoExp]	1,922
33	"Economics, Hospital"[Mesh]	26,299
34	"Economics, Medical"[Mesh:NoExp]	9,312
35	"Economics, Nursing"[Mesh]	4,014
36	"Economics, Pharmaceutical"[Mesh]	3,168
37	economic*[Title/Abstract] OR cost[Title/Abstract] OR costs[Title/Abstract] OR costly[Title/Abstract] OR costing[Title/Abstract]	1,275,349

	OR price[Title/Abstract] OR prices[Title/Abstract] OR pricing[Title/Abstract] OR pharmacoeconomic*[Title/Abstract]	
38	(expenditure*[Title/Abstract] NOT energy[Title/Abstract])	42,823
39	"value for money"[Title/Abstract]	2,385
40	budget*[Title/Abstract]	41,248
41	#29 OR #30 OR #31 OR #33 OR #34 OR #35 OR #36 OR #37 OR #38 OR #39 OR #40	1,437, 375
42	"energy cost"[Title/Abstract] OR "oxygen cost"[Title/Abstract]	5,319
43	"metabolic cost"[Title/Abstract]	2,002
44	"energy expenditure"[Title/Abstract] OR "oxygen expenditure"[Title/Abstract]	32,500
45	#42 OR #43 OR #44	38,590
46	#41 NOT #45	1,428,326
47	letter[Publication Type]	1,310,527
48	editorial[Publication Type]	737,011
49	historical article[Publication Type]	417,127
50	#47 OR #48 OR #49	2,442,622
51	#46 NOT #50	1,383,193
52	Animals[Mesh] NOT Humans[Mesh]	5,380,612
53	#51 NOT #52	1,294,530
54	#28 AND #53	2,576
55	#28 AND #53 from 2015/1/1 - 2025/10/31	1,559

Table S2. PubMed search strategy for PCVs

Search number	Query	Results
1	"Streptococcus pneumoniae"[Mesh]	25,846
2	"Pneumococcal Infections"[Mesh]	23,492
3	"Pneumonia, Pneumococcal"[Mesh]	5,645
4	Pneumococcal disease[Title/Abstract]	5,123
5	Pneumonia[Title/Abstract]	173,566
6	#1 OR #2 OR #3 OR #4 OR #5	203,196
7	"Immunization"[Mesh]	226,965
8	"Immunotherapy, Active"[Mesh:NoExp]	2,523
9	"Immunization Programs"[Mesh:NoExp]	13,928
10	("Vaccination"[Mesh]) OR "Mass Vaccination"[Mesh:NoExp]	121,642
11	vaccine[Title/Abstract] OR vaccination[Title/Abstract] OR immunisation[Title/Abstract] OR immunization[Title/Abstract]	456,554
12	#7 OR #8 OR #9 OR #10 OR #11	538,005
13	#6 AND #12	18,337
14	"Pneumococcal Vaccines"[Mesh]	9,705
15	"Heptavalent Pneumococcal Conjugate Vaccine"[Mesh]	1,288
16	Pneumococcal vaccine[Title/Abstract]	3,595
17	Pneumococcal Conjugate Vaccine[Title/Abstract]	4,432
18	PCV[Title/Abstract] OR PCV10[Title/Abstract] OR PCV-10[Title/Abstract] OR PCV13[Title/Abstract] OR PCV-13[Title/Abstract]	10,687
19	#14 OR #15 OR #16 OR #17 OR #18	19,514
20	#13 OR #19	28,734
21	"Economics"[Mesh:NoExp]	27,552

22	"Costs and Cost Analysis"[Mesh]	282,096
23	"Economics, Dental"[Mesh:NoExp]	1,922
24	"Economics, Hospital"[Mesh]	26,295
25	"Economics, Medical"[Mesh:NoExp]	9,312
26	"Economics, Nursing"[Mesh]	4,014
27	"Economics, Pharmaceutical"[Mesh]	3,168
29	economic*[Title/Abstract] OR cost[Title/Abstract] OR costs[Title/Abstract] OR costly[Title/Abstract] OR costing[Title/Abstract] OR price[Title/Abstract] OR prices[Title/Abstract] OR pricing[Title/Abstract] OR pharmacoeconomic*[Title/Abstract]	1,273,821
30	(expenditure*[Title/Abstract] NOT energy[Title/Abstract])	42,787
31	"value for money"[Title/Abstract]	2,383
32	budget*[Title/Abstract]	41,222
33	#21 OR #22 OR #23 OR #24 OR #25 OR #26 OR #27 OR #29 OR #30 OR #31 OR #32	1,435,774
34	"energy cost"[Title/Abstract] OR "oxygen cost"[Title/Abstract]	5,318
35	"metabolic cost"[Title/Abstract]	1,999
36	"energy expenditure"[Title/Abstract] OR "oxygen expenditure"[Title/Abstract]	32,475
37	#34 OR #35 OR #36	38,561
38	#33 NOT #37	1,426,768
39	letter[Publication Type]	1,309,947
40	editorial[Publication Type]	736,604
41	historical article[Publication Type]	417,060
42	#39 OR #40 OR #41	2,441,571
43	#38 NOT #42	1,381,652
44	Animals[Mesh] NOT Humans[Mesh]	5,379,093
45	#43 NOT #44	1,293,110
46	#20 AND #45	2,172
47	#20 AND #45 from 2015/1/1 - 2025/10/31	1,186

Table S3. PubMed search strategy for RVs

Search number	Query	Results
1	"Rotavirus"[Mesh]	10,374
2	Rotavirus[Title/Abstract]	17,241
3	infant diarrhoea[Title/Abstract] OR infant diarrhea[Title/Abstract]	253
4	infantile diarrhoea[Title/Abstract] OR infantile diarrhea[Title/Abstract]	970
5	#1 OR #2 OR #3 OR #4	19,073
6	"Immunization"[Mesh]	227,163
7	"Immunotherapy, Active"[Mesh:NoExp]	2,523
8	"Immunization Programs"[Mesh:NoExp]	13,946
9	("Vaccination"[Mesh]) OR "Mass Vaccination"[Mesh:NoExp]	121,782
10	vaccine[Title/Abstract] OR vaccination[Title/Abstract] OR immunisation[Title/Abstract] OR immunization[Title/Abstract]	457,070
11	#6 OR 7 OR #8 OR #9 OR #10	538,569
12	#5 AND #11	6,114
13	"Rotavirus Vaccines"[Mesh]	3,056
14	Rotavirus vaccine[Title/Abstract]	2,783

15	Rotavirus vaccines[Title/Abstract]	1,428
16	Rotavirus vaccine[Title/Abstract] OR Rotavirus vaccines[Title/Abstract]	3,256
17	Rotavirus vaccin*[Title/Abstract]	4,077
18	Rotarix[Title/Abstract] OR RotaTeq[Title/Abstract] OR Rota-Teq[Title/Abstract] OR Rotavac[Title/Abstract] OR RotaSiil[Title/Abstract]	867
19	RV1[Title/Abstract] OR RV5[Title/Abstract] OR RIX4414[Title/Abstract]	752
20	#13 OR #17 OR #18 OR #19	5,219
21	#12 OR #20	6,977
22	"Economics"[Mesh:NoExp]	27,552
23	"Costs and Cost Analysis"[Mesh]	282,306
24	"Economics, Dental"[Mesh:NoExp]	1,922
25	"Economics, Hospital"[Mesh]	26,303
26	"Economics, Medical"[Mesh:NoExp]	9,312
27	"Economics, Nursing"[Mesh]	4,014
28	"Economics, Pharmaceutical"[Mesh]	3,168
29	economic*[Title/Abstract] OR cost[Title/Abstract] OR costs[Title/Abstract] OR costly[Title/Abstract] OR costing[Title/Abstract] OR price[Title/Abstract] OR prices[Title/Abstract] OR pricing[Title/Abstract] OR pharmacoeconomic*[Title/Abstract]	1,276,077
30	(expenditure*[Title/Abstract] NOT energy[Title/Abstract])	42,837
31	"value for money"[Title/Abstract]	2,386
32	budget*[Title/Abstract]	41,266
33	#22 OR #23 OR #24 OR #25 OR #26 OR #27 OR #28 OR #29 OR #30 OR #31 OR #32	1,438,137
34	"energy cost"[Title/Abstract] OR "oxygen cost"[Title/Abstract]	5,324
35	"metabolic cost"[Title/Abstract]	2,004
36	"energy expenditure"[Title/Abstract] OR "oxygen expenditure"[Title/Abstract]	32,505
37	#34 OR #35 OR #36	38,602
38	#33 NOT #37	1,429,081
39	letter[Publication Type]	1,310,890
40	editorial[Publication Type]	737,217
41	historical article[Publication Type]	417,165
42	#39 OR #40 OR #41	2,443,226
43	#38 NOT #42	1,383,936
44	Animals[Mesh] NOT Humans[Mesh]	5,381,223
45	#43 NOT #44	1,295,239
46	#21 AND #45	970
47	#21 AND #45 from 2015/1/1 - 2025/10/31	456

Searches on the Tufts CEA Registry and the INAHTA database were conducted by each country of interest, which are the following: Algeria, Egypt, Iran, Jordan, Lebanon, Morocco, Palestine, and Tunisia. The advanced search function of both sources was utilised.

Table S4. Tufts CEA Registry search strategy and results

Search number	Query	Results
1	Algeria “DZ”	23
2	Egypt “DZ”	39

3	Iran "IR"	153
4	Jordan "JO"	15
5	Lebanon "LB"	13
6	Morocco "MA"	25
7	Palestine "PS"	1
8	Tunisia "TS"	18

Date last searched: 31/10/2025

Table S5. International HTA database search strategy and results

Search number	Country	Number of records	HTA organisation	Relevant (Y/N)
1	Algeria	0	N/A	N/A
2	Egypt	0	N/A	N/A
3	Iran	0	N/A	N/A
4	Jordan	0	N/A	N/A
5	Lebanon	0	N/A	N/A
6	Morocco	0	N/A	N/A
7	Palestine	0	N/A	N/A
8	Tunisia	13	National Authority for Assessment and Accreditation in Healthcare (INEAS)	No

Date last searched: 31/10/2025

Supplementary S2: Characteristics of included studies

The following characteristics were extracted from the included papers in this study.

Table S6. General characteristics of included studies

Author	Year published	Study type	Country	Target population	Vaccine	Baseline year of intervention	Intervention and comparator	Study funder
				**Human Papillomavirus vaccine (HPV)**				
Bahr et al	2019	CBA	Lebanon	11-year-old girls	Cervarix™	2016	HPV vaccination campaign vs no vaccination	No funding was received for this study
Bashari et al	2025	CEA	Iran	12-year-old girls	Bivalent HPV vaccine, Quadrivalent HPV vaccine, Nine-valent HPV vaccine	not reported	HPV vaccination vs no vaccination	No funding was received for this study
Gamaoun, R.	2018	CMA	Tunisia	For HPV vaccination: 12-year-old girls	Bivalent HPV vaccine	2017	National HPV vaccination programme vs cervical cancer screening using the Pap smear test	No funding was received for this study
Hagens et al	2024	CEA	Iran	Men and women across 16 age groups	HPV vaccine	2020	HPV vaccination vs no vaccination	No funding was received for this study
Jit et al	2014	CEA	179 countries including Algeria, Egypt, Iran, Jordan, Lebanon, Morocco, Tunisia	12-year-old girls	HPV 16/18	2012-2013	HPV16/18 versus no further vaccine introductions; HPV16/18 versus increased vaccine introductions in 2012-2032	World Health Organization
Khatibi et al	2014	CEA	Iran	15-year-old females	HPV-16 and 18 (Gardasil)	2013	HPV vaccination vs no vaccination	No funding was received for this study

Khiari et al	2024	CEA	Tunisia	10-84-year-old females	HPV 16/18	Not reported	HPV 16/18 vaccination vs no vaccination; combined HPV vaccination and screening vs screening alone	No funding was received for this study
Laraj et al	2025	CEA	Tunisia	12-year-old girls	Cecolin, Cervarix, Gardasil-4, Gardasil-9	2025	HPV vaccination vs no vaccination	Bill & Melinda Gates Foundation and Vaccine Impact Modelling Consortium (VIMC)
Messoudi et al	2019	CEA	Morocco	Women	HPV 16/18	Not reported	Three strategies modelled: 1) Screening women (30-49 years old) versus no intervention 2) HPV16/18 vaccination of girls at least 14 years old versus no intervention 3) Combined vaccination and screening versus screening alone	Instituto de Salud Carlos III, European Regional Development Fund
Rosettie et al	2021	Cost-effectiveness and meta-regression analysis	195 countries including Iran and Egypt	Not reported	HPV quadrivalent and bivalent vaccines	Not reported	HPV vaccination vs no vaccination	Bill & Melinda Gates Foundation
Sargazi et al	2022	CBA	Iran	9-14-year-old girls	HPV bivalent and quadrivalent vaccine	2020	HPV vaccination vs no vaccination	Tehran University of Medical Sciences
Yaghoubi et al	2018	CEA	Iran	9-year-old females	HPV-16 and 18 (Gardasil)	2015	HPV vaccination vs no vaccination	World Health Organization
				**Pneumococcal Conjugate Vaccine (PCV)**				

Ezoji et al	2019	CEA	Iran	Children under 5 years old	PCV13	2014	PCV vaccination vs no vaccination	ProVac International Working Group, which is funded entirely by a grant from the Bill & Melinda Gates Foundation
Lagoubi et al	2022	CEA	Tunisia	Entire population (a specific birth cohort was used applied to the entire population)	PCV13 or PhiD-CV	Not reported	PCV vaccination vs no vaccination	GlaxoSmithKline Biologicals SA
Pugh et al	2018	CEA	Algeria, Tunisia	Children under 2 years old	PCV10, PCV13	2016	PCV13 or PCV10 vs no vaccination	Pfizer
Sevilla et a	2022	CUA and CBA	Egypt	100 successive birth cohorts eligible for vaccination in the year of birth	PCV10 (SynflorixTM), PCV13 (Prevenar13TM)	2016	PCV13 or PCV10 vs no vaccination; PCV13 vs PCV10	Pfizer
Sibak et al	2015	CEA	Egypt	Children under 5 years old	PCV13	2013	PCV vaccination vs no vaccination	Grant from the Bill and Melinda Gates Foundation to the PAHO
				**Rotavirus vaccine (RV)**				
Azad et al	2019	BIA	Iran	Population based on birth from March 2018 to February 2023	RotaTeq® (RV5)	2018	RotaTeq vs No immunisation	Tehran University of Medical Sciences
Debellut et al	2020	CEA	Palestine	Children under 5 years old	Rotarix®, Rotavac®	2016	Rotarix vs no vaccination; Rotavac vs no vaccination; Rotavac vs Rotarix	Bill & Melinda Gates Foundation
Debellut et al	2021	CEA and benefit-risk analysis	63 MICs, including Algeria, Egypt, Iran, Jordan, Lebanon, Morocco, Palestine, Tunisia	Children under 5 years old	Rotarix®, Rotavac®, Rotasiil® and next-generation rotavirus vaccines (NGRVs)	2020	Current rotavirus vaccines vs no vaccination; NGRVs vs current rotavirus vaccines	Bill & Melinda Gates Foundation

Debellut et al	2022	CEA	137 LMICs, including Iran, Egypt, Algeria, Tunisia, Jordan, Lebanon, Morocco, Palestine	Children under 5 years old	Licensed vaccines: Rotavac®, Rotasiil®, Rotarix®, Other vaccines: RV3-BB, trivalent P2-VP8, trivalent P2-VP8 comprising part of a DTP-containing combination vaccine	2025	Each vaccine was comapred to no vaccination and to each other	Bill & Melinda Gates Foundation
Javanbakht et al	2015	CEA	Iran	Children 1–59 months old	RotaTeq® (RV5)	2014	Rotavirus vaccination vs no vaccination	ProVac International Working Group, which is funded entirely by a grant from the Bill & Melinda Gates Foundation
Mohy et al	2024	CEA	Morocco	Children under 5 years old	HRV, HBRV, BRV-PV	2023	Rotavirus vaccination vs no vaccination	GlaxoSmithKline Biologicals SA, Costello Medical
Mousavi Jarrahi et al	2015	CEA	Iran	Children under 5 years old	Rotarix®	2009	Rotavirus vaccination vs no vaccination	Vaccine Preventable Diseases, Communicable Disease Control Dept., Ministry of Health, I. R. of Iran.
Paternina-Caicedo et al	2015	CEA	LMICs including Algeria, Egypt, Jordan, Morocco, Tunisia	Children under 5 years old	RV1, RV5	2010	RV1 or RV5 vaccination vs no vaccination	Universidad Nacional de Colombia and Universidad de Cartagena
Shakerian et al	2015	CEA	Iran	Children under 5 years old	RotaTeq®, Rotarix®	Not reported	RotaTeq® vs no vaccination; Rotarix® vs no vaccination	Iran National Institute of Health Research

Supplementary S3: Included cost inputs

The table below shows the cost inputs retrieved from the included studies. They are organised by disease of interest, namely, human papillomavirus (HPV), pneumococcal virus (PCV) , and rotavirus (RV).

Table S7. Included costs

Author	Study type	Included costs	Direct medical costs	Direct costs included	Indirect costs reported (Yes/No)
			**Human Papillomavirus**		
Bahr et al	CBA	Direct medical costs<br>Vaccination costs	Cervical cancer management costs	Invasive case (cervical cancer): USD 3,916<br>Non-invasive case (cervical cancer): USD 2,524	No
Bashari et al	CEA	Direct medical costs<br>Vaccination costs	Screening costs<br>Diagnostic costs<br>Treatment of HPV-related diseases	HPV test: USD 65<br>PAP Smear test: USD 14.75<br>Colposcopy: USD 83.35<br>Conization: USD 213.92<br>Cervical cancer stage I: USD 4,712.74<br>Cervical cancer stage II: USD 7,497.89<br>Cervical cancer stage III: USD 9,025.21<br>Cervical cancer stage IV: USD 8,004.63<br>Follow-up cost: USD 160.21<br>GW Treatment: USD 85.63	No
Gamoun, R	CMA	Direct medical costs<br>Vaccination costs	Cost of medical care for an incidence of cervical cancer	Incremental annual cost (Cervical cancer): USD 1,664 (4,149 Tunisian dinars)	No
Hagens et al	CEA	Direct medical costs<br>Vaccination costs	Hospitalisation costs<br>Costs of prescribing drugs<br>Cervical cancer treatment costs	USD 26,850	No

Jit et al	CEA	Direct medical costs<br>Vaccination costs	Cancer treatment costs	Not reported	No
Khatibi et al	CEA	Direct medical costs<br>Vaccination costs	Treatment costs for HPV-related diseases	Cervical cancer: IRR80,000,000<br>Genital wart: IRR2,500,000<br>CIN I: IRR5,000,000<br>CIN II: IRR10,000,000<br>CIN III: IRR30,000,000	No
Khiari et al	CEA	Direct medical costs<br>Vaccination costs	Screening costs<br>Diagnostic costs<br>Cancer treatment costs	Screening test (cytology): USD 36.7<br>Diagnostic (colposcopy): USD 36.6<br>Diagnostic (Biopsy): USD 27.1<br>CIN 1 treatment: USD 63.7<br>CIN 2/3 treatment: USD 136.7<br>Cervical cancer stage I and II treatment: USD 1,576<br>Cervical cancer stage III treatment: USD 2,603<br>Cervical cancer stage IV treatment: USD 1,800	No
Laraj et al	CEA	Direct medical costs<br>Vaccination costs<br>Indirect costs	Diagnostic costs<br>Cancer treatment costs (surgical, chemotherapy, radiotherapy, and palliative care for each stage	Diagnostic costs not provided<br><br>Cancer care treatment cost per case (govenrment perspective):<br>Localised stage: USD 2,603<br>Distant stage: USD 1,800<br>Regional stage: USD 750<br>Average cancer treatment cost per case: USD 2,445.14<br><br>Cancer care treatment cost per case (societal perspective):<br>Localised stage: USD 4,055<br>Distant stage: USD 3,252	Yes

				Regional stage: USD 2,202<br>Average cancer treatment cost per case: USD 3,897.14	
Messoudi et al	CEA	Direct medical costs<br>Vaccination costs	Screening costs<br>Diagnostic costs<br>Costs of treatment of precancerous lesion	Screening test (VIA): USD 36.7<br>Diagnostic (Colposcopy): USD 36.6<br>Diagnostic (Biopsy): USD 27.1<br>CIN treatment for CIN 1: USD 63.7<br>CIN treatment for CIN 2/3: USD 136.7<br>Cervical cancer stage I & II: USD 1,026.9<br>Cervical cancer stage III: USD 2,083<br>Cervical cancer stage IV: USD 1,508.5	No
Rosettie et al	Cost-effectiveness and meta-regression analysis	Vaccination costs	Not applicable	Not applicable	No
Sargazi et al	CBA	Direct medical costs<br>Non-direct medical costs<br>Indirect costs<br>Vaccination costs	Diagnostic costs<br>Cancer treatment cost (surgery, chemotherapy, radiotherapy, brachytherapy)<br>Medication costs (Chemotherapy medications)<br>Follow-up costs<br>Transportation costs<br>Indirect costs (cervical cancer)<br>Loss of productivity due to disability<br>Productivity lost due to premature death	Direct medical cost: USD 7,629<br>Diagnostic procedures: USD 146<br>Surgery: USD 1,429<br>Chemotherapy: USD 23<br>Direct medical costs:<br>Radiotherapy: USD 62<br>Brachytherapy: USD 1,417<br>Chemo radiation therapy: USD 3,089<br>Chemotherapy medications: USD 1,228<br>Follow-up: USD 234<br><br>Direct non-medical costs (Transportation cost): USD 190	Yes

Yaghoubi et al	CEA	Direct medical costs<br>Out-of-pocket medical costs<br>Direct non-medical costs<br>Indirect costs<br>Vaccination costs	Government perspective:<br>Public hospital medical costs<br>Social security hospital costs<br>Private hospital costs<br><br>Societal perspective:<br>Out-of-pocket costs for the patient (Household costs)	Direct medical costs and out-of-pocket costs<br>Local cancer:<br>Government cost per treated woman: USD 1,775<br>Household cost per treated woman: USD 1,595<br><br>Regional cancer:<br>Government cost per treated woman: USD 4,775<br>Household cost per treated woman: USD 2,574<br><br>Direct non-medical costs<br>Transportation cost: USD 11.80 per journey<br>Local cancer: 5 trips<br>Regional cancer: 36 trips	Yes
			**Pneumococcal virus**		
Ezoji et al	CEA	Direct medical costs<br>Vaccination costs	Government perspective:<br>Outpatient costs (Acute otitis media, pneumococcal pneumonia, pneumococcal meningitis, pneumococcal NPNM)<br>Inpatient costs (Acute otitis media, pneumococcal pneumonia, pneumococcal meningitis, pneumococcal NPNM)<br>Cost of pneumonia meningitis sequelae<br><br>Household perspective (includes out-of-pocket costs):	Government perspective (outpatient costs per visit):<br>AOM: USD 2.6<br>Pneumococcal pneumonia: USD 7.4<br>Pneumococcal meningitis: USD 10.7<br>Pneumococcal NPNM: USD 15.4<br><br>Government perspective (inpatient costs per visit):<br>Pneumococcal pneumonia: USD 126.2<br>Pneumococcal meningitis: USD 434.4<br>Pneumococcal NPNM: USD 441.5<br><br>Household perspective (outpatient costs per visit):<br>AOM: USD 1.6	No

			Outpatient costs (Acute otitis media, pneumococcal pneumonia, pneumococcal meningitis, pneumococcal NPNM)<br>Inpatient costs (Acute otitis media, pneumococcal pneumonia, pneumococcal meningitis, pneumococcal NPNM)<br>Cost of pneumonia meningitis sequelae	Pneumococcal pneumonia: USD 2.9<br>Pneumococcal meningitis: USD 4.6<br>Pneumococcal NPNM: USD 6.6<br><br>Household perspective (inpatient costs per visit):<br>Pneumococcal pneumonia: USD 117.2<br>Pneumococcal meningitis: USD 128.5<br>Pneumococcal NPNM: USD 100.2<br><br>Government cost of meningitis sequelae per year:<br>Major sequelae (single): 0<br>Major sequelae (multiple): 0<br><br>Household cost of meningitis sequelae per year:<br>Major sequelae (single): USD 4,525.95<br>Major sequelae (multiple): USD 4,525.95	
Lagoubi et al	CEA	Direct medical costs<br>Vaccination costs	Outpatient costs per acute episode<br>Inpatient costs per acute episode<br>Annual sequelae costs (Pneumococcal meningitis)	AOM outpatient cost (GP consultation): USD 43<br>Pneumonia outpatient cost: USD 120<br>Pneumonia inpatient cost: USD 624<br>Pneumococcal bactaeremia inpatient cost: USD 750<br>Pneumococcal meningitis: USD 1,873<br>Pneumococcal meningitis sequelae per year cost: USD 5,985	No
Pugh et al	CUA and CBA	Direct medical costs<br>Vaccination costs	Outpatient costs (Pneumonia, Otitis media)<br>Inpatient costs (Pneumonia, Pneumococcal bactaeremia, NPNM)	Outpatient costs (Otitis media):<br>Algeria: USD 3,205<br>Tunisia: USD 42<br><br>Outpatient cost (Pneumonia):<br>Algeria: USD 8,406<br>Tunisia: USD 118	No

				Inpatient cost (Pneumonia):<br>Algeria: USD 55, 594<br>Tunisia: USD 616<br><br>Inpatient cost (Meningitis):<br>Algeria: USD 166,782<br>Tunisia: USD 1,849<br><br>Inpatient cost (Pneumococcal bactaeremia):<br>Algeria: USD 66,713<br>Tunisia: USD 740	
Sevilla et al	CEA	Direct medical costs<br>Vaccination costs	Direct medical care costs per care episode<br>Outpatient cost (Pneumonia)<br>Inpatient cost (Pneumonia)	Direct medical care costs per care episode:<br>Acute otitis media: USD 1.816 (Range: USD 1.569-2.119)<br>Meningitis: USD 240.415 (Range: USD 201.33-245.89)<br>Bactaeremia: USD 96.834 (Range: USD 87.24-106.92)<br><br>Outpatient cost per care episode (Pneumonia):<br>USD 2.785 (Range: USD 2.612-3.53)<br><br>Inpatient cost per care episode (Pneumonia):<br>USD 31.414 (USD 28.262-33.836)	No
Sibak et al	CEA	Direct medical costs<br>Vaccination costs	Inpatient costs (Pneumococcal pneumonia, Pneumococcal meningitis, NPNM, and all-cause acute otitis media)<br>Outpatient costs (Pneumococcal pneumonia, Pneumococcal	Outpatient costs (Otitis media): USD 4.08 (USD 3.67-4.49)<br>Outpatient cost (Pneumonia): USD 3.91 (USD 3.86-4.72)<br>Outpatient cost (Meningitis): USD 4.81 (USD 3.86-4.72)	No

			meningitis, NPNM, and all-cause acute otitis media)	Outpatient cost (NPNM): USD 4.36 (USD 3.92-4.79)<br><br>Inpatient cost (Pneumonia): USD 147 (USD 138-152)<br>Inpatient cost (Meningitis): USD 270 (USD 328-263)<br>Inpatient cost (NPNM): USD 145 (USD 138-152)	
			**Rotavirus**		
Azad et al	BIA	Direct medical costs<br>Vaccination costs	Inpatient costs<br>Outpatient costs (including prescription and physician visits)	Weighted average outpatient cost: USD 139.72 (Range: USD 107.95-171.49)<br>Weighted average inpatient cost: USD 158.59 (Range: USD 121.83-195.35)	Not applicable
Debellut et al 2020	CEA	Direct medical costs<br>Vaccination costs<br>Indirect costs	Outpatient costs<br>Inpatient costss<br>Indirect outpatient cost<br>Indirect inpatient cost	Outpatient cost for Rotavirus gastroenteritis: USD 7.63<br>Inpatient cost for Rotavirus gastroenteritis: USD 173.85	Yes
Debellut et al 2021	CEA and benefit-risk analysis	Direct medical costs<br>Direct non-medical costs<br>Indirect costs<br>Vaccination costs	Inpatient costs (Rotavirus gastroenteritis)<br>Outpatient costs (Rotavirus gastroenteritis)<br>Sequelae costs (intussusception)	Government perspective (Direct medical costs):<br>Inpatient costs:<br>Algeria: USD 131<br>Egypt: USD 76<br>Iran: USD 210<br>Jordan: USD 111<br>Lebanon: USD 542<br>Morocco: USD 130<br>Palestine: USD 75<br>Tunisia: USD 161<br><br>Outpatient costs:	Yes

Algeria: USD 9
Egypt: USD 7
Iran: USD 10
Jordan: USD 8
Lebanon: USD 22
Morocco: USD 9
Palestine: USD 7
Tunisia: USD 10

Sequelae costs:
Algeria: USD 269
Egypt: USD 243
Iran: USD 560
Jordan: USD 182
Lebanon: USD 248
Morocco: USD 87
Palestine: USD 141
Tunisia: USD 250

Societal perspective (Direct medical + Direct non-medical + indirect costs):
Inpatient costs:
Algeria: USD 169
Egypt: USD 98
Iran: USD 269
Jordan: USD 145
Lebanon: USD 676
Morocco: USD 166
Palestine: USD 99
Tunisia: USD 204

Outpatient costs:
Algeria: USD 14

				Egypt: USD 11<br>Iran: USD 18<br>Jordan: USD 14<br>Lebanon: USD 35<br>Morocco: USD 15<br>Palestine: USD 12<br>Tunisia: USD 15<br><br>Sequelae costs:<br>Algeria: USD 342<br>Egypt: USD 316<br>Iran: USD 705<br>Jordan: USD 256<br>Lebanon: USD 367<br>Morocco: USD 133<br>Palestine: USD 198<br>Tunisia: USD 331	
Debellut et al 2022	CEA	Direct medical costs<br>Direct non-medical costs<br>Indirect costs<br>Vaccination costs	Inpatient costs (Rotavirus gastroenteritis)<br>Outpatient costs/ clinic visits (Rotavirus gastroenteritis)<br>Sequelae costs (intussusception)	Inpatient costs:<br>Algeria: USD 169.41<br>Egypt: USD 98.08<br>Iran: USD 268.84<br>Lebanon: USD 675.87<br>Morocco: USD 165.75<br>Palestine: USD 98.92<br>Tunisia: USD 203.53<br>Jordan: USD 145.04<br><br>Outpatient costs:<br>Algeria: USD 14.38<br>Egypt: USD 10.53<br>Iran: USD 17.74<br>Lebanon: USD 35.45	Yes (costs presented summed up direct and indirect costs)

				Morocco: USD 14.97<br>Palestine: USD 11.65<br>Tunisia: USD 15.15<br>Jordan: USD 14.01	
Javanbakht et al	CEA	Direct medical costs<br>Out-of-pocket medical costs<br>Vaccination costs	Government perspective:<br>Outpatient costs(Rotavirus non-severe, severe)<br>Inpatient costs (rotavirus non-severe, severe)<br><br>Household perspective (out-of-pocket costs):<br>Outpatient costs(Rotavirus non-severe, severe)<br>Inpatient costs (rotavirus non-severe, severe)	Government cost per outpatient visit:<br>Rotavirus (non-severe) cases: USD 2.23<br>Rotavirus (severe) cases: USD 4.69<br><br>Government cost per inpatient admission:<br>Rotavirus (severe) cases: USD 174.52<br><br>Household cost per outpatient visit:<br>Rotavirus (non-severe) cases: USD 3.85<br>Rotavirus (severe) cases: USD 6.60<br><br>Household cost per inpatient admission:<br>Rotavirus (severe) cases: USD 45.29	No
Mohy et al	CEA	Direct medical costs<br>Vaccination costs<br>Caregiver productivity costs	Outpatient costs<br>Inpatient costs	Cost of RVGE outpatient cost: USD 11<br><br>Cost of RVGE hospitalization: USD 156	Yes
Mousavi Jarrahi et al	CEA	Direct medical costs<br>Direct non-medical costs<br>Indirect costs<br>Vaccination costs	Inpatient costs<br>Outpatient costs<br><br>Direct non-medical costs:<br>Transportation costs	Hospitalization cost: USD 214<br>Out-patient visit cost: USD 16<br><br>Direct non-medical costs:<br>Transportation costs (hospitalized): USD 11<br>Transportation costs (outpatient): USD 4	Yes

Paternina-Caicerdo et al	CEA	Direct medical costs<br>Vaccination costs	Cost of bed per day<br>Cost of clinic visits	The study did not report values for the cost per bed-day or the cost of clinic visits.	No
Shakerian et al	CEA	Direct medical costs<br>Indirect costs<br>Vaccination costs	Inpatient costs (private and public hospitals)<br>Outpatient costs (consultations, medications, and out-of-pocket care)	Did not present costs in the study	Yes

Table S8. Vaccine costs included

Study	Number of doses	Vaccine unit	Vaccine cost and currency	Supplier	Wastage rate (vaccines)	Delivery cost	Handling costs	Injection supply costs
				**Human Papillomavirus**				
Bahr et al	2 (no booster doses mentioned)	Price per dose	USD 71	Manufacturer	Not reported	Not reported	Not reported	Not reported
Bashari et al	2 and 3 (no booster doses mentioned)	Price per dose	Bivalent: USD 41.32<br>Quadrivalent: USD 88.45<br>Nine-valent: USD 75.71	Bivalent and Nine-valent: estimated from proxy country (Greece)<br>Quadrivalent: Iran Food and Drug Administration	Not reported	Not reported	Not reported	Not reported
Gamoun, R	2	Price per dose	USD 50<br>USD 4.50	Manufacturer<br>GAVI	Not reported	Not reported	Not reported	Not reported

	(no booster doses mentioned)							
Hagens et al	2 (no booster doses mentioned)	Price per fully vaccinated child	USD 152	Not reported (obtained from a previous study in Iran)	Not reported	Administration cost: USD 1	Not reported	Not reported
Jit et al	3 (no booster doses mentioned)	Price per fully vaccinated child	Algeria: USD 55.52<br>Egypt: USD 55.47<br>Iran: USD 55.40<br>Jordan: USD 55.25<br>Lebanon: USD 54.79<br>Morocco: USD 55.55<br>Tunisia: USD 55.05	Not reported (cited costs come from original studies)	Not reported	Not reported	Not reported	Not reported
Khatibi et al	3 (no booster doses mentioned)	Price per dose	Euro 85.5	Based on the proposed price of the vaccine in Iran	Not reported	Not reported	Not reported	Not reported
Khiari et al	2 (no booster doses mentioned)	Price per dose	USD 10<br>(Range: USD 15-45)	Directory of Pharmacy and Medicine in Tunisia	Not reported	Administration cost: USD 5	Not reported	Not reported
Laraj et al	1 (no booster doses mentioned)	Price per dose	Gardasil-9: USD 25.00<br>Cecolin: USD 3.65<br>Cervarix: USD 10.25<br>Gardasil-4: USD 14.14	Gardasil-9: MI4A/V3P vaccine purchase data<br>Cecolin: Tunisian Ministry of Health<br>Cervarix: UNICEF	5%	10%	3%	USD 0.07 per dose and<br>USD 1.30 per box of 100 syringes

				Gardasil-4: Not reported				
Messoudi et al	2 (no booster doses mentioned)	Price per dose	USD 10 (Range: USD 10-15)	PAHO Revolving Fund price	Not reported	Administration cost: USD 5	Not reported	Not reported
Rosettie et al	Not reported	Price per dose	HICs: USD 52.42<br>UMICs: USD 40.13<br>LMICs: USD 4.50	LMICs price: UNICEF UMICs and HICs price: WHO's Market Information for Access to Vaccines (MI4A)	Not reported	Not reported	Not reported	Not reported
Sargazi et al	2 (no booster doses mentioned)	Price per fully vaccinated child	Bivalent: USD 29<br>Quadrivalent: USD 151	Not reported (Expert opinion)	Not reported	Not reported	Not reported	Not reported
Yaghoubi et al	2 and 3 (no booster doses mentioned)	Price per fully vaccinated child	2-dose vaccination: USD 15.87<br>3-dose vaccination: USD 13.75	Behestan Darou (local pharmaceutical company)	5%	2%	3%	Not reported
				**Pneumococcal virus**				
Ezoji et al	3 (no booster doses mentioned)	Price per dose	USD 20 (Range: USD 14.24-35.00)	Pfizer	5%	2%	3%	Not reported

Lagoubi et al	3 (no booster doses mentioned)	Price per dose	PHiD-CV: USD 12.85<br>PCV 13: USD 14.50	PHiD-CV: 2020 PAHO Revolving Fund<br>PCV 13: PAHO	10%	Administration cost: USD 1	Not reported	Not reported
Pugh et al	2 (1 booster dose included)	Price per dose	PCV-10:<br>Algeria: USD 14.60<br>Tunisia: USD 14.60<br><br>PCV-13:<br>Algeria: USD 15.70<br>Tunisia: USD 15.70	Not reported (Expert opinion)	Not reported	Administration fee:<br>Algeria: USD 0.92<br>Tunisia: USD 2.48	Not reported	Not reported
Sevilla et al	2 (1 booster dose included)	Price per dose	PCV-10: USD 12.85<br>PCV-13: USD 14.50	2019 Pan American Health Organization Revolving Fund vaccine prices	1%	1%	3%	Not reported
Sibak et al	3 (no booster doses mentioned)	Price per dose	USD 14.24	Vacsera (local manufacturer)	1%	1%	3%	Safety boxes (80 syringes per box): $0.60<br>Administering syringe per dose: $0.06
**Rotavirus**								
Azad et al	Not reported	Price per dose<br>Price per fully vaccinated child	Per dose: USD 7<br>Per patient: USD 21.21	Iranian Ministry of Health	5% (1-5%)	Not reported	Not reported	Not reported

Debellut et al 2020	ROTAVAC: 3<br>ROTARIX: 2<br>(no booster doses mentioned)	Price per dose	Per dose:<br>ROTAVAC: USD 1<br>ROTARIX: USD 4	ROTAVAC: GAVI and UNICEF<br>ROTARIX: UNICEF	ROTAVAC: 4.7%<br>ROTARIX: 0.3%	ROTAVAC: USD 0.0249<br>ROTARIX: USD 0.0264	3.50%	Not reported
Debellut et al 2021	ROTAVAC: 3<br>ROTARIX: 2<br>ROTASIIL: 2<br>(no booster doses mentioned)	Price per fully vaccinated child<br>Price per dose	ROTARIX:<br>Algeria: USD 25<br>Egypt: USD 26<br>Iran: USD 23<br>Jordan: USD 25<br>Lebanon: USD 16<br>Morocco: USD 26<br>Palestine: USD 26<br>Tunisia: USD 25<br><br>ROTAVAC:<br>Algeria: USD 10<br>Egypt: USD 11<br>Iran: USD 8<br>Jordan: USD 9<br>Lebanon: USD -3<br>Morocco: USD 10<br>Palestine: USD 10<br>Tunisia: USD 10<br><br>ROTASIIL:<br>Algeria: USD 8<br>Egypt: USD 9<br>Iran: USD 6<br>Jordan: USD 7<br>Lebanon: USD -5	GSK, Bharat Biotech and Serum Institute (Manufacturers)	ROTARIX: 5%<br>ROTAVAC: 23%<br>ROTASIIL: 10%	USD 2.05	Handling cost: 3.5%<br><br>International freight/transport cost: 6%	Not reported

			Morocco: USD 8<br>Palestine: USD 9<br>Tunisia: USD 8<br><br>Manufacturer vaccine price per dose:<br>ROTARIX: USD 10.25<br>Rotavac: USD 1.25<br>Rotassil: USD 1					
Debellut et al 2022	Not reported	Price per dose	ROTAVAC:<br>Algeria: USD 1.25<br>Egypt: USD 1.25<br>Iran: USD 1.25<br>Lebanon: USD 1.25<br>Morocco: USD 1.25<br>Palestine: USD 1.25<br>Tunisia: USD 1.25<br>Jordan: USD 1.25<br><br>ROTARIX:<br>Algeria: USD 14.77<br><br>Egypt: USD 14.77<br>Iran: USD 14.77<br>Lebanon: USD 14.77<br>Morocco: USD 14.77<br><br>Palestine: USD 14.77<br>Tunisia: USD 14.77<br>Jordan: USD 14.77<br><br>ROTASIIL:	GAVI	Rotarix: 4%<br>Rotavac: 10%<br>Rotasiil: 4%	USD 1.33 (low income)<br>USD 2.05 (lower and upper middle income)	International handling: 3%<br>International transportation: 6%	Not reported

			Algeria: USD 1.63<br>Egypt: USD 1.63<br>Iran: USD 1.63<br>Lebanon: USD 1.63<br>Morocco: USD 1.63<br>Palestine: USD 1.63<br>Tunisia: USD 1.63<br>Jordan: USD 1.63					
Javanbakht et al	3 (no booster doses mentioned)	Price per dose	USD 10 (Range: USD 7-12)	Merck and Co. Inc. (Manufacturer)	5%	2%	3%	Not reported
Mohy et al	HRV: 2<br>HBRV: 3<br>BRV-PV: 1<br>(no booster doses mentioned)	Price per dose	HRV: USD 6.50<br>HBRV: USD 5.20<br>BRV-PV: USD 3.00	PAHO Revolving Fund price	4%	Not reported	0.35%	Not reported
Mousavi Jarrahi et al	2 (no booster doses mentioned)	Price per fully vaccinated child	USD 10	Unspecified local manufacturer	5%	Not reported	Not reported	Not reported
Paternina-Caicerdo et al	RV1: 2<br>RV 5: 3<br>(no booster doses mentioned)	Price per dose<br>Price per fully vaccinated child	RV1:<br>Price per dose: USD 6.88<br>Price per patient: USD 13.76<br><br>RV5:<br>Price per dose: USD 5.25	PAHO Revolving Fund price	10%	Not reported	Not reported	Not reported

			Price per patient: USD 15.75					
Shakerian et al	RotaTeq: 3<br>Rotarix: 2<br>(no booster doses mentioned)	Price per dose	RotaTeq: USD 7<br>Rotarix: USD 7	Ministry of Health	5%	Not reported	Not reported	Not reported

Supplementary S4: Main outcomes

The following characteristics were extracted from the included papers in this study.

Table S9. Main outcomes from included studies

Study	Study type	Perspective	Health outcome	Main outcome	Main findings<br>Base case ICER	Main conclusions
Bahr et al	CBA	Implied perspective: governmental payer (National Treasury)	No health outcome	Cost of vaccination	USD5,407,790 to vaccinate 38,083 11-year-old girls	No health outcome
Bashari et al	CEA	Payer	QALYs gained, cervical cancer death reduction, cervical cancer cases reduction	ICER	2-doses:<br>Bivalent= USD33,179 /QALY<br>Quadrivalent= USD45,088 /QALY<br>Nine-valent= USD50,067 /QALY<br><br>3-doses:<br>Bivalent= USD50,264 /QALY	None of the vaccination strategies were considered cost-effective

					Quadrivalent= USD69,384 /QALY Nine-valent= USD76,778 /QALY	
Gamaoun, R.	CMA	Implied perspective: health system	Cervical cancer cases avoided	Incremental cost per avoided case	The ascending incremental costs by avoided cervical cancer case are: 1- the national vaccination programme through the GAVI support (USD1803), 2- the cervical cancer screening according to 10-year periodicity (USD8219), 3- the cervical cancer screening according to 5-year periodicity (USD14,567), 4- the cervical cancer screening according to 3-year periodicity (USD20,479), 5- and finally the national vaccination programme according to the manufacturer marketed price (USD36,854).	The anti-HPV national vaccination program combined with cervical cancer screening according to 5-year periodicity present the best cost-effective strategy for cervical cancer prevention.
Hagens et al	CEA	Healthcare system	QALYs gained, deaths averted	ICER	Vaccinating boys and girls: USD7,916 /QALY Vaccinating only girls: USD4,949 /QALY Vaccinating only boys: USD15,529 /QALY	Vaccinating girls only was found to be cost-effective, with an ICER close to the GDP per capita. Vaccinating both sexes was shown to be less cost-effective compared to girls only, and vaccinating boys only was not found to be cost-effective, with an ICER between once and

						three times, and greater than three times the GDP per capita, respectively.
Jit et al	CEA	Implied perspective: healthcare or purchaser	Deaths prevented, cancers prevented, DALYs averted	Cost per life year saved	Cost per life year saved:<br>Algeria: USD4,250<br>Egypt: USD13,900<br>Iran: USD10,800<br>Jordan: USD11,700<br>Lebanon: USD6,760<br>Morocco: USD1,700<br>Tunisia: USD6,500<br><br>Cost per DALY prevented:<br>Algeria: USD 3,830<br>Egypt: USD 12,600<br>Iran: USD 9,940<br>Jordan: USD 10,800<br>Lebanon: USD 6,180<br>Morocco: USD 1,560<br>Tunisia: USD 5,970	HPV vaccination is likely to be very cost effective in most countries and cost-effective in almost every country in the world.
Khatibi et al	CEA	Governmental	QALYs gained	ICER	Base-case: IRR 439,092,468 /QALY	The quadrivalent HPV vaccine (Gardasil) is not cost-effective in Iran based on the base-case parameters value.
Khiari et al	CEA	Not reported	QALYs gained	ICER	Combined vaccine and screening strategy vs no intervention: USD966.5 /QALY<br>Combined vaccine and screening strategy vs screening alone: USD2,293.3 /QALY	Compared with screening alone, the implementation of HPV vaccination in addition to the current cytology screening programme in Tunisia would be considered cost-effective on the basis of the threshold GDP per capita.

Laraj et al	CEA	Governmental and societal	DALYs averted, deaths averted, cases averted	ICER	**Cecolin**<br>PRIME model:<br>Government perspective: USD162 /DALY<br>Societal perspective: USD34 /DALY<br><br>UNIVAC model:<br>Government perspective: USD121 /DALY<br>Societal perspective: USD13 /DALY<br><br>**Cervarix**<br>PRIME model:<br>Government perspective: USD845 /DALY<br>Societal perspective: USD718 /DALY<br><br>UNIVAC model:<br>Government perspective: USD625 /DALY<br>Societal perspective: USD516 /DALY<br><br>**Gardasil-4**<br>PRIME model:<br>Government perspective: USD1,266 /DALY<br>Societal perspective: USD1,139 /DALY	The four HPV vaccines (Cecolin, Cervarix Gardasil-4, and Gardasil-9) were cost-effective in the Tunisian context.

UNIVAC model:
Government perspective: USD935 /DALY
Societal perspective: USD827 /DALY

Gardasil-9
PRIME model:
Government perspective: USD1,845 /DALY
Societal perspective: USD1,718 /DALY

UNIVAC model:
Government perspective: USD1,362 /DALY
Societal perspective: USD1,253 /DALY

Cecolin (cross protection)
PRIME model:
Government perspective: USD151 /DALY
Societal perspective: USD24 /DALY

UNIVAC model:
Government perspective: USD113 /DALY
Societal perspective: USD5 /DALY

					**Cervarix (cross protection)**<br>PRIME model:<br>Government perspective: USD686 /DALY<br>Societal perspective: USD559 /DALY<br><br>UNIVAC model:<br>Government perspective: USD507 /DALY<br>Societal perspective: USD399 /DALY	
Messoudi et al	CEA	Healthcare system	Years of life saved (YLS)	ICER	**Strategy: Visual inspection with acetic acid 5%**<br>ICER for vaccinating 14-year-old girls: dominated<br>ICER for vaccination + screening: USD2,327 /YLS<br><br>**Strategy: Visual inspection with acetic acid 15%**<br>ICER for vaccinating 14-year-old girls: dominated<br>ICER for vaccination + screening: USD2,911 /YLS<br><br>**Strategy: Visual inspection with acetic acid 20%**<br>ICER for vaccinating 14-year-old girls: USD1,150 /YLS<br>ICER for vaccination + screening: USD3,743 /YLS	HPV vaccination could be highly effective and cost-effective in Morocco. Current screening would be good value for money compared with no intervention, but scaling-up screening coverage would make it inefficient compared with vaccination.

					**Strategy: Visual inspection with acetic acid 30%**<br>ICER for vaccinating 14-year-old girls: USD1,150 /YLS<br>ICER for vaccination + screening: USD5,308 /YLS<br><br>**Strategy: Visual inspection with acetic acid 50%**<br>ICER for vaccinating 14-year-old girls: USD1,150 /YLS<br>ICER for vaccination + screening: USD10,712 /YLS<br><br>**Strategy: Visual inspection with acetic acid 70%**<br>ICER for vaccinating 14-year-old girls: USD1,150 /YLS<br>ICER for vaccination + screening: USD 14,170 /YLS<br><br>**Strategy: Visual inspection with acetic acid 100%**<br>ICER for vaccinating 14-year-old girls: USD1,150 /YLS<br>ICER for vaccination + screening: USD20,167 /YLS	
Rosettie et al	Cost-effectiveness and meta-	Healthcare payer	DALYs averted	ICER	Algeria: USD6,369 (1,169 to 20,202) /DALY averted<br>Egypt: USD10,057 (1,854 to 31,337) /DALY averted	The authors concluded that their results identified countries where the HPV vaccine is a good value, but whether the vaccine is cost-effective based on the

	regression analysis				Iran: USD9,222 (1,683 to 28,936) /DALY averted<br>Jordan: USD10,438 (1,921 to 32,502) /DALY averted<br>Lebanon: USD4,196 (1,246 to 15,793) /DALY averted<br>Morocco: USD5,317 (979 to 16,942) /DALY averted<br>Palestine: USD9,632 (1,773 to 30,034) /DALY averted	country-specific predicted ICER and cost-effectiveness threshold is not reported.
Sargazi et al	CBA	Societal	No units of benefit (costs only)	Cost-benefit ratio	Cost-benefit ratio (willingness to pay approach):<br>Bivalent: -USD15.11<br>Quadrivalent: USD2.51<br><br>Cost-benefit ratio (cost of illness approach):<br>Bivalent: USD258.12<br>Quadrivalent: USD43.51	This study confirmed the benefits of the national bivalent and quadrivalent vaccination programs. Specifically, the bivalent vaccine's benefit is higher than its cost under the cost-of-illness method; the quadrivalent vaccine's benefit is higher than its cost under both evaluation methods (cost-of-illness and willingness to pay).
Yaghoubi et al	CEA	Governmental and societal	DALYs averted, cases averted, deaths averted	ICER	Governmental: USD15,205 /DALY<br>Societal: USD14,999 /DALY	Introducing a three-dose HPV vaccination program is currently not cost-effective in Iran.
			**Pneumococcal conjugate vaccine (PCV)**			
Ezoji et al	CEA	Governmental and societal	DALYs averted, cases averted, deaths averted, hospital admissions averted,	ICER	Government perspective: USD1,890 /DALY<br>Societal perspective: USD1,538 /DALY	Introduction of PCV-13 for children under 5 years in the Islamic Republic of Iran would be cost-effective

			outpatient visits averted			
Lagoubi et al	CEA	Payer	QALYs gained, deaths averted, cases averted, medical visits averted, hospitalisations averted	ICUR	PCV13: dominated<br>PhiD-CV: USD484 /QALY	PCVs are a cost-effective strategy to relieve the burden associated with diseases caused by S. pneumoniae and NTHi in Tunisia. PHiD-CV is more cost-effective than PCV13, generating similar health benefits, at a reduced net cost of almost $1 million USD per vaccinated cohort.
Pugh et al	CEA	Payer	QALYs gained, deaths averted, cases averted	ICER	PCV13 in Tunisia: USD848 /QALY<br>PCV10 in Tunisia: USD1366 /QALY<br>PCV13 in Algeria: USD308 /QALY<br>PCV10 in Algeria: USD731 /QALY	PCV NIPs are highly cost-effective, highly impactful public health interventions.
Sevilla et a	CUA and CBA	Societal (CBA) and Payer (CUA)	QALYs gained, deaths averted, cases averted, hospitalisations averted	ICER	PCV13 vs. no program: USD925.6 (512.08–1,734.9) /QALY<br><br>PCV10 vs. no program: USD1,984.41 (1,186.32–3,804.67) /QALY<br><br>PCV13 vs. PCV10: USD173.98 (87.59–331.23) /QALY	A universal pediatric PCV13 program represents good value for money for policymakers in Egypt.
Sibak et al	CEA	Governmental	DALYs averted, deaths averted, cases averted, inpatient	ICER	USD 3,916 /DALY	PCV would be cost-effective from the government perspective

			admissions averted, outpatient visits averted			
				**Rotavirus vaccine (RV)**		
Azad et al	BIA	Healthcare system	Outpatient cases averted, inpatient cases averted	Budget impact	Incremental cost during 5 years of immunisation: USD 131,450,210	The inclusion of rotavirus vaccine in the national vaccination program would have a significant effect on health budgets and would raise government expenditure.
Debellut et al 2020	CEA	Healthcare system and societal	DALYs averted, cases averted, deaths averted, outpatient visits averted, hospitalisations averted	ICER	ROTARIX compared to no vaccine (health system perspective): USD1,254 /DALY<br>ROTARIX compared to no vaccine (societal perspective): Cost-saving (-USD794 /DALY)<br>ROTAVAC compared to no vaccine (health system perspective): USD353 /DALY<br>ROTAVAC compared to no vaccine (societal perspective): Cost-saving (-USD1,695 /DALY)<br>ROTAVAC compared to ROTARIX (health system perspective): Cost-saving (-USD901 /DALY)<br>ROTAVAC compared to	From the health system perspective, rotavirus vaccination with either ROTARIX or ROTAVAC is a cost-effective intervention in Palestine compared to no vaccination. When accounting for averted healthcare-related costs for households, using either vaccine is a cost-saving intervention. When evaluating the switch, ROTAVAC presents an economic advantage over ROTARIX and shifting from ROTARIX to ROTAVAC was a cost-saving option from both the health system and societal perspectives.

					ROTARIX (societal perspective): Cost-saving (-USD901 /DALY)	
Debellut et al 2021	CEA and benefit-risk analysis	Governmental and societal	DALYs averted, cases averted, deaths averted, outpatient visits averted, hospitalisations averted	ICER	**Government perspective:**<br>Rotarix:<br>Algeria: USD8,332 (3,520–15,992) /DALY<br>Egypt: USD2,936 (2,081–3,872) /DALY<br>Iran: USD6,669 (3,839–9,417) /DALY<br>Jordan: USD9,497 (5,799–14,016) /DALY<br>Lebanon: USD6,374 (2,052–10,352) /DALY<br>Morocco: USD3,502 (2,271–4,970) /DALY<br>Palestine: USD10,171 (5,609–16,294) /DALY<br>Tunisia: USD11 335 (6,271–17,834) /DALY<br><br>Rotavac:<br>Algeria: USD2,781 (1,024–5,418) /DALY<br>Egypt: USD1,048 (657–1,370) /DALY<br>Iran: USD1,625 (0–2,767) /DALY<br>Jordan: USD3,318 (1,671–5,003) /DALY<br>Lebanon: CS (CS–1,690)	In most MICs not eligible for Gavi funding, rotavirus vaccination has high probability to be cost-effective with a favourable benefit–risk profile.

/DALY
Morocco: USD1,140 (643–1,637) /DALY
Palestine: USD3,604 (1,803–5,835) /DALY
Tunisia: 3,324 (1,298–5,587) /DALY

Rotasil:
Algeria: USD2,278 (789–4,530) /DALY
Egypt: USD871 (511–1,160) /DALY
Iran: USD1,198 (0–2,257) /DALY
Jordan: USD2,701 (1,223–4,195) /DALY
Lebanon: CS (CS–1,029) /DALY
Morocco: USD937 (491–1,361) /DALY
Palestine: USD2,986 (1,430–4,927) /DALY
Tunisia: USD2,636 (841–4,626) /DALY

Societal perspective:

Rotarix:
Algeria: USD8,071 /DALY
Egypt: USD2,869 /DALY
Iran: USD6,172 /DALY
Jordan: USD9,106 /DALY

					Lebanon: USD4,905 /DALY<br>Morocco: USD3,410 /DALY<br>Palestine: USD9,893 /DALY<br>Tunisia: USD10,842 /DALY<br><br>Rotavac:<br>Algeria: USD2,520 /DALY<br>Egypt: USD982 /DALY<br>Iran: USD1,128 /DALY<br>Jordan: USD2,927 /DALY<br>Lebanon: cost saving<br>Morocco: USD1,048 /DALY<br>Palestine: USD3,326 /DALY<br>Tunisia: USD2,831 /DALY<br><br>Rotasil:<br>Algeria: USD2,017 /DALY<br>Egypt: USD805 /DALY<br>Iran: USD701 /DALY<br>Jordan: USD2,310 /DALY<br>Lebanon: cost saving<br>Morocco: USD845 /DALY<br>Palestine: USD2,708 /DALY<br>Tunisia: USD2,143 /DALY	
Debellut et al 2022	CEA	Societal	DALYs averted, cases averted, deaths averted, outpatient visits averted, hospitalisations averted	ICER	ROTAVAC<br>Algeria: USD2,662/ DALY<br>Egypt: USD1,093 /DALY<br>Iran: USD1,400 /DALY<br>Lebanon: cost-saving<br>Morocco: USD1,271 /DALY<br>Palestine: USD3,385 /DALY<br>Tunisia: USD3,286 /DALY	The results show that while currently available live oral rotavirus vaccines (LORVs) remain a good investment for countries and donors today, an injectable next-generation rotavirus vaccine (iNGRV) with comparable or superior efficacy to LORVs is likely to be cost-effective in the majority of LMICs.

					Jordan: USD3,138 /DALY<br><br>ROTASIIL<br>Algeria: USD3,011 /DALY<br>Egypt: USD1,221 /DALY<br>Iran: USD1,725 /DALY<br>Lebanon: cost-saving<br>Morocco: USD1,429 /DALY<br>Palestine: USD3,795 /DALY<br>Tunisia: USD3,790 /DALY<br>Jordan: 3,566 /DALY<br><br>ROTARIX<br>Algeria: USD12,148 /DALY<br>Egypt: USD4,507 /DALY<br>Iran: USD10,063 /DALY<br>Lebanon: USD8,802 /DALY<br>Morocco: USD5,498 /DALY<br>Palestine: USD14,395 /DALY<br>Tunisia: USD16,788 /DALY<br>Jordan: USD14,647 /DALY	
Javanbakht et al	CEA	Healthcare system and societal	DALYs averted, cases averted, deaths averted, outpatient visits averted, inpatient admissions averted	ICER	Government perspective: USD 2,868 /DALY<br>Society perspective: USD 382 /DALY	Introduction of rotavirus vaccine is a highly cost-effective strategy from the government perspective.
Mohy et al	CEA	Country payer and societal	QALYs gained	ICER	Base case ICER (HRV as the reference) country payer perspective<br>HBRV: HRV is dominant	HRV was associated with lower costs versus HBRV from both the country payer (−$1.8M) and societal (−$4.1M) perspectives, and versus BRV-PV 1-dose

					BRV-PV: USD328,376<br><br>Base case ICER (HRV as the reference) societal perspective<br>HBRV: HRV is dominant<br>BRV-PV: HRV is dominant	vial from the societal perspective (−$187,000), dominating those options in the cost-effectiveness analysis. However, costs of BRV-PV 1-dose vial were lower than HRV from the payer perspective, resulting in an ICER of approximately $328,376 per QALY, above the assumed cost effectiveness threshold of $3,500. Vaccination with a 2-dose schedule of HRV may be a cost-saving option and could lead to better health outcomes for children in Morocco versus 3-dose schedule rotavirus vaccines.
Mousavi Jarrahi et al	CEA	Healthcare system and societal	DALYs averted, deaths averted, outpatient visits averted, hospitalisations averted	ICER	A cost of 19 USD for each DALY averted from the health care system perspective<br>A saving of 278 USD for each DALY averted from the societal perspective	Introducing rotavirus vaccine into EPI program would be highly cost-effective public health intervention in Iran.
Paternina-Caicedo et al	CEA	Healthcare system	DALYs averted, deaths averted	ICER	Not reported by country	Rotavirus vaccine is cost-effective in most analysed countries.
Shakerian et al	CEA	Healthcare system	DALYs averted, cases averted	ICER	ICER RotaTeq:<br>3,672 episodes per 100,000 with USD10 price in base year= USD16,186 /DALY<br>6,243 episodes per 100,000 with SD10 price in base year= USD15,376 /DALY<br>36,000 episodes per 100,000 with USD10 price in base	Assuming that the illness episodes are 100% and 300% for Rotarix and 300% for RotaTeq, the ratio of cost per DALY averted is highly cost-effective.

year= USD9,582 /DALY<br>108,000 episodes per 100,000 with USD10 price in base year= USD3,701 /DALY<br>3,672 episodes per 100,000 with USD15 price in base year= USD23,380 /DALY<br>6,243 episodes per 100,000 with USD15 price in base year= USD22,278 /DALY<br>36,000 episodes per 100,000 with USD15 price in base year= USD14,443 /DALY<br>108,000 episodes per 100,000 with USD15 price in base year= USD6,444 /DALY<br><br>ICER Rotarix:<br>3,672 episodes per 100,000 with USD10 price in base year= USD9,402 /DALY<br>6,243 episodes per 100,000 with USD10 price in base year= USD8,868 /DALY<br>36,000 episodes per 100,000 with USD10 price in base year= USD5,000 /DALY<br>108,000 episodes per 100,000 with USD10 price in base year= USD1,115 /DALY<br>3,672 episodes per 100,000 with USD15 price in base year= USD13,599 /DALY

	6,243 episodes per 100,000 with USD15 price in base year= USD12,895 /DALY<br>36,000 episodes per 100,000 with USD15 price in base year= USD7,835 /DALY<br>108,000 episodes per 100,000 with USD15 price in base year= USD2,715 /DALY

Supplementary S5: ICERs utilised in the study

The ICERs gathered from the included studies were converted to the same currency (USD) and was expressed as a ratio relative to GDP per capita based on the USD conversion using the corresponding year.

Table S10. ICERs used in the study

Study	ICER (Details)	ICER (Value)	Per QALY/DALY/ Life year	Country	Currency	Year	GDP per capita	ICER as a ratio to GDP per capita
				**Human papillomavirus (HPV)**				
Bahr et al				Lebanon	USD	2016	8089.012088	Does not apply
Bashari et al	2-dose bivalent vaccine	33,179	Per QALY	Iran	USD	2019	3997.493545	8.30
	2-dose quadrivalent vaccine	45,088	Per QALY	Iran	USD	2019	3997.493545	11.28
	2-dose nine-valent vaccine	50,067	Per QALY	Iran	USD	2019	3997.493545	12.52

	3-dose bivalent vaccine	50,264	Per QALY	Iran	USD	2019	3997.493545	12.57
	3-dose quadrivalent vaccine	69,384	Per QALY	Iran	USD	2019	3997.493545	17.36
	3-dose nine-valent vaccine	76,778	Per QALY	Iran	USD	2019	3997.493545	19.21
Hagens et al	Vaccinating boys and girls	7916	Per QALY	Iran	USD	2020	3202.500035	2.47
	Vaccinating only girls	4,949	Per QALY	Iran	USD	2020	3202.500035	1.55
	Vaccinating only boys	15,529	Per QALY	Iran	USD	2020	3202.500035	4.85
Jit et al		4250	Cost per life year saved	Algeria	USD	2011	5916.313644	0.72
		13,900	Cost per life year saved	Egypt	USD	2011	2590.643849	5.37
		10,800	Cost per life year saved	Iran	USD	2011	8025.687434	1.35
		11,700	Cost per life year saved	Jordan	USD	2011	3946.855039	2.96
		6,760	Cost per life year saved	Lebanon	USD	2011	7834.962571	0.86
		1,700	Cost per life year saved	Morocco	USD	2011	3301.98877	0.51

		6,500	Cost per life year saved	Tunisia	USD	2011	4420.647722	1.47
		3,830	Cost per DALY prevented	Algeria	USD	2011	5916.313644	0.65
		12,600	Cost per DALY prevented	Egypt	USD	2011	2590.643849	4.86
		9,940	Cost per DALY prevented	Iran	USD	2011	8025.687434	1.24
		10,800	Cost per DALY prevented	Jordan	USD	2011	3946.855039	2.74
		6,180	Cost per DALY prevented	Lebanon	USD	2011	7834.962571	0.79
		1,560	Cost per DALY prevented	Morocco	USD	2011	3301.98877	0.47
		5,970	Cost per DALY prevented	Tunisia	USD	2011	4420.647722	1.35
Khatibi et al		23,845	Per QALY	Iran	USD	2013	6222.41948	3.83
Khiari et al	Combined vaccine and screening strategy vs no intervention	966.5	Per QALY	Tunisia	USD	2020	3548.653637	0.27

	Combined vaccine and screening strategy vs screening alone	2,293.30	Per QALY	Tunisia	USD	2020	3548.653637	0.65
Laraj et al	Cecolin (PRIME model) government perspective	162	Per DALY	Tunisia	USD	2024	4181.137893	0.04
	Cecolin (PRIME model) societal perspective	34	Per DALY	Tunisia	USD	2024	4181.137893	0.01
	Cecolin (UNIVAC model) government perspective	121	Per DALY	Tunisia	USD	2024	4181.137893	0.03
	Cecolin (UNIVAC model) societal perspective	13	Per DALY	Tunisia	USD	2024	4181.137893	0.00
	Cervarix (PRIME model) government perspective	845	Per DALY	Tunisia	USD	2024	4181.137893	0.20
	Cervarix (PRIME model) societal perspective	718	Per DALY	Tunisia	USD	2024	4181.137893	0.17
	Cervarix (UNIVAC model)	625	Per DALY	Tunisia	USD	2024	4181.137893	0.15

government perspective							
Cervarix (UNIVAC model) societal perspective	516	Per DALY	Tunisia	USD	2024	4181.137893	0.12
Gardasil-4 (PRIME model) government perspective	1,266	Per DALY	Tunisia	USD	2024	4181.137893	0.30
Gardasil-4 (PRIME model) societal perspective	1,139	Per DALY	Tunisia	USD	2024	4181.137893	0.27
Gardasil-4 (UNIVAC model) government perspective	935	Per DALY	Tunisia	USD	2024	4181.137893	0.22
Gardasil-4 (UNIVAC model) societal perspective	827	Per DALY	Tunisia	USD	2024	4181.137893	0.20
Gardasil-9 (PRIME model) government perspective	1,845	Per DALY	Tunisia	USD	2024	4181.137893	0.44
Gardasil-9 (PRIME model) societal perspective	1,718	Per DALY	Tunisia	USD	2024	4181.137893	0.41
Gardasil-9 (UNIVAC	1,362	Per DALY	Tunisia	USD	2024	4181.137893	0.33

model) government perspective							
Gardasil-9 (UNIVAC model) societal perspective	1,253	Per DALY	Tunisia	USD	2024	4181.137893	0.30
Cecolin cross-protection (PRIME model) government perspective	151	Per DALY	Tunisia	USD	2024	4181.137893	0.04
Cecolin cross-protection (PRIME model) societal perspective	24	Per DALY	Tunisia	USD	2024	4181.137893	0.01
Cecolin cross-protection (UNIVAC model) government perspective	113	Per DALY	Tunisia	USD	2024	4181.137893	0.03
Cecolin cross-protection (UNIVAC model) societal perspective	5	Per DALY	Tunisia	USD	2024	4181.137893	0.00
Cervarix cross-protection (PRIME model) government perspective	686	Per DALY	Tunisia	USD	2024	4181.137893	0.16

	Cervarix cross-protection (PRIME model) societal perspective	559	Per DALY	Tunisia	USD	2024	4181.137893	0.13
	Cervarix cross-protection (UNIVAC model) government perspective	507	Per DALY	Tunisia	USD	2024	4181.137893	0.12
	Cervarix cross-protection (UNIVAC model) societal perspective	399	Per DALY	Tunisia	USD	2024	4181.137893	0.10
Messoudi et al	Strategy: Visual inspection with acetic acid 5% ICER for vaccinating 14-year-old girls	Dominated	Per YLS	Morocco	USD	2018	3501.697998	cost saving*
	Strategy: Visual inspection with acetic acid 5% ICER for vaccination + screening	2,327	Per YLS	Morocco	USD	2018	3501.697998	0.66
	Strategy: Visual inspection with acetic acid 15%	Dominated	Per YLS	Morocco	USD	2018	3501.697998	cost saving*

ICER for vaccinating 14-year-old girls							
Strategy: Visual inspection with acetic acid 15% ICER for vaccination + screening	2,911	Per YLS	Morocco	USD	2018	3501.697998	0.83
Strategy: Visual inspection with acetic acid 20% ICER for vaccinating 14-year-old girls	1,150	Per YLS	Morocco	USD	2018	3501.697998	0.33
Strategy: Visual inspection with acetic acid 20% ICER for vaccination + screening	3,743	Per YLS	Morocco	USD	2018	3501.697998	1.07
Strategy: Visual inspection with acetic acid 30% ICER for vaccinating 14-year-old girls	1,150	Per YLS	Morocco	USD	2018	3501.697998	0.33
Strategy: Visual inspection with acetic acid 30% ICER for vaccination + screening	5,308	Per YLS	Morocco	USD	2018	3501.697998	1.52

Strategy: Visual inspection with acetic acid 50% ICER for vaccinating 14-year-old girls	1,150	Per YLS	Morocco	USD	2018	3501.697998	0.33
Strategy: Visual inspection with acetic acid 50% ICER for vaccination + screening	10,712	Per YLS	Morocco	USD	2018	3501.697998	3.06
Strategy: Visual inspection with acetic acid 70% ICER for vaccinating 14-year-old girls	1,150	Per YLS	Morocco	USD	2018	3501.697998	0.33
Strategy: Visual inspection with acetic acid 70% ICER for vaccination + screening	14,170	Per YLS	Morocco	USD	2018	3501.697998	4.05
Strategy: Visual inspection with acetic acid 100% ICER for vaccinating 14-year-old girls	1,150	Per YLS	Morocco	USD	2018	3501.697998	0.33
Strategy: Visual inspection with acetic acid 100%	20,167	Per YLS	Morocco	USD	2018	3501.697998	5.76

	ICER for vaccination + screening							
Rosettie et al		6,369	Per DALY averted	Algeria	USD	2017	4554.66754	1.40
		10,057	Per DALY averted	Egypt	USD	2017	2395.103333	4.20
		9,222	Per DALY averted	Iran	USD	2017	6000.933464	1.54
		10,438	Per DALY averted	Jordan	USD	2017	4065.616287	2.57
		4,196	Per DALY averted	Lebanon	USD	2017	8608.21085	0.49
		5,137	Per DALY averted	Morocco	USD	2017	3296.526611	1.56
		9,632	Per DALY averted	Palestine	USD	2017	3620.360487	2.66
Yaghoubi et al	Government perspective	15,205	Per DALY	Iran	USD	2015	4952.733555	3.07
	Societal perspective	14,999	Per DALY	Iran	USD	2015	4952.733555	3.03
				**Pneumococcal Virus (PCV)**				
Ezoji et al	Government perspective	1,890	Per DALY	Iran	USD	2014	5672.063729	0.33
	Societal perspective	1,538	Per DALY	Iran	USD	2014	5672.063729	0.27
Lagoubi et al	PCV13	Dominated	Per QALY	Tunisia	USD	2020	3548.653637	
	PhiD-CV	484	Per QALY	Tunisia	USD	2020	3548.653637	0.14
Pugh et al	PCV13	848	Per QALY	Tunisia	USD	2016	3847.803544	0.22
	PCV10	1,366	Per QALY	Tunisia	USD	2016	3847.803544	0.36
	PCV13	308	Per QALY	Algeria	USD	2016	4424.98529	0.07

	PCV10	731	Per QALY	Algeria	USD	2016	4424.98529	0.17
Sevilla et al	PCV13 vs. no program	925.6	Per QALY	Egypt	USD	2016	3270.628777	0.28
	PCV10 vs. no program	1,984.41	Per QALY	Egypt	USD	2016	3270.628777	0.61
	PCV13 vs. PCV10	173.98	Per QALY	Egypt	USD	2016	3270.628777	0.05
Sibak et al		3,916	Per DALY	Egypt	USD	2013	3025.525636	1.29
				**Rotavirus (RV)**				
Debellut et al 2020	ROTARIX compared to no vaccine (health system perspective)	1,254	Per DALY	Palestine	USD	2018	3562.330943	0.35
	ROTARIX compared to no vaccine (societal perspective): Cost-saving	-794	Per DALY	Palestine	USD	2018	3562.330943	cost saving*
	ROTAVAC compared to no vaccine (health system perspective)	353	Per DALY	Palestine	USD	2018	3562.330943	0.10
	ROTAVAC compared to no vaccine (societal	-1,695	Per DALY	Palestine	USD	2018	3562.330943	cost saving*

	perspective): Cost-saving							
	ROTAVAC compared to ROTARIX (health system perspective): Cost-saving	-901	Per DALY	Palestine	USD	2018	3562.330943	cost saving*
	ROTAVAC compared to ROTARIX (societal perspective): Cost-saving	-901	Per DALY	Palestine	USD	2018	3562.330943	cost saving*
Debelut et al 2021	Rotarix (government perspective)	8,332	Per DALY	Algeria	USD	2018	4577.210292	1.82
		2,936	Per DALY	Egypt	USD	2018	2484.703156	1.18
		6,669	Per DALY	Iran	USD	2018	4783.010673	1.39
		9,497	Per DALY	Jordan	USD	2018	4145.439897	2.29
		6,374	Per DALY	Lebanon	USD	2018	9174.536662	0.69
		3,502	Per DALY	Morocco	USD	2018	3501.697998	1.00
		10,171	Per DALY	Palestine	USD	2018	3562.330943	2.86
		11,335	Per DALY	Tunisia	USD	2018	3628.103665	3.12
	Rotavac (government perspective)	2,781	Per DALY	Algeria	USD	2018	4577.210292	0.61
		1,048	Per DALY	Egypt	USD	2018	2484.703156	0.42
		1,625	Per DALY	Iran	USD	2018	4783.010673	0.34
		33,180	Per DALY	Jordan	USD	2018	4145.439897	8.00
		cost saving*	Per DALY	Lebanon	USD	2018	9174.536662	cost saving*
		1,140	Per DALY	Morocco	USD	2018	3501.697998	0.33

	3,604	Per DALY	Palestine	USD	2018	3562.330943	1.01
	3,324	Per DALY	Tunisia	USD	2018	3628.103665	0.92
Rotasiil (Government perspective)	2,278	Per DALY	Algeria	USD	2018	4577.210292	0.50
	871	Per DALY	Egypt	USD	2018	2484.703156	0.35
	1,198	Per DALY	Iran	USD	2018	4783.010673	0.25
	2,701	Per DALY	Jordan	USD	2018	4145.439897	0.65
	cost saving*	Per DALY	Lebanon	USD	2018	9174.536662	cost saving*
	937	Per DALY	Morocco	USD	2018	3501.697998	0.27
	2,986	Per DALY	Palestine	USD	2018	3562.330943	0.84
	2,636	Per DALY	Tunisia	USD	2018	3628.103665	0.73
Rotarix (societal perspective)	2,278	Per DALY	Algeria	USD	2018	4577.210292	0.50
	871	Per DALY	Egypt	USD	2018	2484.703156	0.35
	1,198	Per DALY	Iran	USD	2018	4783.010673	0.25
	2,701	Per DALY	Jordan	USD	2018	4145.439897	0.65
	cost saving*	Per DALY	Lebanon	USD	2018	9174.536662	cost saving*
	937	Per DALY	Morocco	USD	2018	3501.697998	0.27
	2,986	Per DALY	Palestine	USD	2018	3562.330943	0.84
	2,636	Per DALY	Tunisia	USD	2018	3628.103665	0.73
Rotarix (societal perspective)	8,071	Per DALY	Algeria	USD	2018	4577.210292	1.76
	2,869	Per DALY	Egypt	USD	2018	2484.703156	1.15
	6,172	Per DALY	Iran	USD	2018	4783.010673	1.29
	9,106	Per DALY	Jordan	USD	2018	4145.439897	2.20
	4,905	Per DALY	Lebanon	USD	2018	9174.536662	0.53
	3,410	Per DALY	Morocco	USD	2018	3501.697998	0.97
	9,893	Per DALY	Palestine	USD	2018	3562.330943	2.78
	10,482	Per DALY	Tunisia	USD	2018	3628.103665	2.89
	2,520	Per DALY	Algeria	USD	2018	4577.210292	0.55

	Rotavac (societal perspective)	982	Per DALY	Egypt	USD	2018	2484.703156	0.40
		1,128	Per DALY	Iran	USD	2018	4783.010673	0.24
		2,297	Per DALY	Jordan	USD	2018	4145.439897	0.55
		cost saving*	Per DALY	Lebanon	USD	2018	9174.536662	cost saving*
		1,048	Per DALY	Morocco	USD	2018	3501.697998	0.30
		3,326	Per DALY	Palestine	USD	2018	3562.330943	0.93
		2,831	Per DALY	Tunisia	USD	2018	3628.103665	0.78
	Rotasiil (societal perspective)	2,017	Per DALY	Algeria	USD	2018	4577.210292	0.44
		805	Per DALY	Egypt	USD	2018	2484.703156	0.32
		701	Per DALY	Iran	USD	2018	4783.010673	0.15
		2,310	Per DALY	Jordan	USD	2018	4145.439897	0.56
		cost saving*	Per DALY	Lebanon	USD	2018	9174.536662	cost saving*
		845	Per DALY	Morocco	USD	2018	3501.697998	0.24
		2,708	Per DALY	Palestine	USD	2018	3562.330943	0.76
		2,143	Per DALY	Tunisia	USD	2018	3628.103665	0.59
Debellut et al 2022	Rotavac	2,662	Per DALY	Algeria	USD	2018	4577.210292	0.58
		1,093	Per DALY	Egypt	USD	2018	2484.703156	0.44
		1,400	Per DALY	Iran	USD	2018	4783.010673	0.29
		cost saving*	Per DALY	Lebanon	USD	2018	9174.536662	cost saving*
		1,271	Per DALY	Morocco	USD	2018	3501.697998	0.36
		3,385	Per DALY	Palestine	USD	2018	3562.330943	0.95
		3,286	Per DALY	Tunisia	USD	2018	3628.103665	0.91
		3,138	Per DALY	Jordan	USD	2018	4145.439897	0.76
	Rotasiil	3,011	Per DALY	Algeria	USD	2018	4577.210292	0.66
		1,221	Per DALY	Egypt	USD	2018	2484.703156	0.49
		1,725	Per DALY	Iran	USD	2018	4783.010673	0.36
		cost saving*	Per DALY	Lebanon	USD	2018	9174.536662	cost saving*

		1,429	Per DALY	Morocco	USD	2018	3501.697998	0.41
		3,795	Per DALY	Palestine	USD	2018	3562.330943	1.07
		3,790	Per DALY	Tunisia	USD	2018	3628.103665	1.04
		3,566	Per DALY	Jordan	USD	2018	4145.439897	0.86
		3,011	Per DALY	Algeria	USD	2018	4577.210292	0.66
	Rotarix	12,148	Per DALY	Algeria	USD	2018	4577.210292	2.65
		4,507	Per DALY	Egypt	USD	2018	2484.703156	1.81
		10,063	Per DALY	Iran	USD	2018	4783.010673	2.10
		8,802	Per DALY	Lebanon	USD	2018	9174.536662	0.96
		5,498	Per DALY	Morocco	USD	2018	3501.697998	1.57
		14,395	Per DALY	Palestine	USD	2018	3562.330943	4.04
		16,788	Per DALY	Tunisia	USD	2018	3628.103665	4.63
		14,647	Per DALY	Jordan	USD	2018	4145.439897	3.53
Javanbakht et al	Government perspective	2,868	Per DALY	Iran	USD	2013	6222.741948	0.46
	Societal perspective	382	Per DALY	Iran	USD	2013	6222.741948	0.06
Mohy et al	Base case ICER (HRV as the reference) country payer perspective for HBRV	HRV is dominant	Per QALY gained	Morocco	USD	2022	3463.137695	cost saving*
	Base case ICER (HRV as the reference) country payer perspective for BRV-PV	328,376	Per QALY gained	Morocco	USD	2022	3463.137695	94.82
	Base case ICER (HRV as the	HRV is dominant	Per QALY gained	Morocco	USD	2022	3463.137695	cost saving*

	reference) societal perspective for HBRV							
	Base case ICER (HRV as the reference) societal perspective for BRV-PV	HRV is dominant	Per QALY gained	Morocco	USD	2022	3463.137695	cost saving*
Mousavi Jarrahi et al	Healthcare system perspective	19	Per DALY averted	Iran	USD	2009	5415.522806	0.00
	Societal perspective	A saving of USD 278	Per DALY averted	Iran	USD	2009	5415.522806	cost saving*
Shakerian et al	3,672 episodes per 100,000 with USD10 price in base year (RotaTeq)	16,186	Per DALY	Iran	USD	2010	6291.194437	2.57
	6,243 episodes per 100,000 with SD10 price in base year (RotaTeq)	15,376	Per DALY	Iran	USD	2010	6291.194437	2.44
	36,000 episodes per 100,000 with USD10 price in base year (RotaTeq)	9,582	Per DALY	Iran	USD	2010	6291.194437	1.52

108,000 episodes per 100,000 with USD10 price in base year (RotaTeq)	3,701	Per DALY	Iran	USD	2010	6291.194437	0.59
3,672 episodes per 100,000 with USD15 price in base year (RotaTeq)	23,380	Per DALY	Iran	USD	2010	6291.194437	3.72
6,243 episodes per 100,000 with USD15 price in base year (RotaTeq)	22,278	Per DALY	Iran	USD	2010	6291.194437	3.54
36,000 episodes per 100,000 with USD15 price in base year (RotaTeq)	14,443	Per DALY	Iran	USD	2010	6291.194437	2.30
108,000 episodes per 100,000 with USD15 price in base year (RotaTeq)	6,444	Per DALY	Iran	USD	2010	6291.194437	1.02
3,672 episodes per 100,000 with USD10 price in base year (Rotarix)	9402	Per DALY	Iran	USD	2010	6291.194437	1.49
6,243 episodes per 100,000 with USD10 price in	8,868	Per DALY	Iran	USD	2010	6291.194437	1.41

base year (Rotarix)							
36,000 episodes per 100,000 with USD10 price in base year (Rotarix)	5,000	Per DALY	Iran	USD	2010	6291.194437	0.79
108,000 episodes per 100,000 with USD10 price in base year (Rotarix)	1,115	Per DALY	Iran	USD	2010	6291.194437	0.18
3,672 episodes per 100,000 with USD15 price in base year (Rotarix)	13,599	Per DALY	Iran	USD	2010	6291.194437	2.16
6,243 episodes per 100,000 with USD15 price in base year (Rotarix)	12,895	Per DALY	Iran	USD	2010	6291.194437	2.05
36,000 episodes per 100,000 with USD15 price in base year (Rotarix)	7,835	Per DALY	Iran	USD	2010	6291.194437	1.25
108,000 episodes per 100,000 with USD15 price in base year (Rotarix)	2,715	Per DALY	Iran	USD	2010	6291.194437	0.43

Note: *Strategy that dominates the comparator: lower cost and greater health benefits

Supplementary S6: Quality assessment scoring

This section highlights the full JBI and full CHEERS item-by-item scoring leading to the combined score presented in the main study.

Table S11. Full JBI item-by-item scoring

Study	P1	P2	P3	P4	P5	P6	P7	P8	P9	P10	P11	P12
Bahr et al	2	2	1	Not applicable	2	Not applicable	1	Not applicable	2	0	2	2
Bashari et al	2	2	2	2	2	2	2	2	2	2	2	2
Gamaoun	2	2	2	2	2	2	2	2	2	1	2	0
Hagens et al	2	2	2	2	2	2	2	1	2	1	2	0
Jit et al	2	2	2	2	2	2	2	2	2	1	2	2
Khatibi et al	2	2	2	2	2	2	2	2	2	1	2	2
Khiari et al	2	2	2	2	2	2	2	2	2	1	2	2
Laraj et al	2	2	2	2	2	2	2	2	2	2	2	2
Messoudi et al	2	2	2	2	2	2	2	2	2	1	2	2
Rosettie et al	2	1	2	2	2	2	2	2	2	1	2	2
Sargazi et al	2	2	2	Not applicable	2	Not applicable	2	Not applicable	2	1	2	2
Yaghoubi et al	2	2	2	2	2	2	2	2	2	1	2	2
Ezoji et al	2	2	2	2	2	2	2	2	2	1	2	1
Lagoubi et al	2	2	2	2	2	2	2	2	2	2	2	2
Pugh et al	2	2	2	2	2	2	2	2	2	1	2	2
Sevilla et a	2	2	2	2	2	2	2	2	2	2	2	2
Sibak et al	2	2	2	2	2	2	2	2	2	1	2	2
Azad et al	2	2	2	2	2	2	2	2	2	1	2	2
Debellut 2020	2	2	2	2	2	2	2	1	2	2	2	2
Debellut 2021	2	2	2	2	2	2	2	2	2	2	2	2
Debellut 2022	2	2	2	2	2	2	2	2	2	2	2	2
Javanbakht et al	2	2	2	2	2	2	2	2	2	1	2	2
Mohy et al	2	2	2	2	2	2	2	2	2	2	2	2

Mousavi Jarrahi et al	2	2	2	2	2	2	2	2	2	1	2	2
Paternina-Caicedo et al	2	2	2	2	2	2	2	2	2	2	2	2

Parameters: P1: Research question clearly stated; P2: Relevant comparators identified; P3: Relevant costs identified; P4: Relevant outcomes identified; P5: Costs measured accurately; P6: Outcomes measured accurately; P7: Valuation of costs credible; P8: Valuation of outcomes credible; P9: Assumptions clearly stated; P10: Uncertainty analysis appropriate; P11: Conclusions justified by results; P12: Generalisability to target setting considered

Table S12. Full CHEERS item-by-item scoring

Study	P1	P2	P3	P4	P5	P6	P7	P8	P9	P10	P11	P12	P13	P14	P15	P16	P17
Bahr et al	2	2	2	2	2	0	Not applicable	2	1	Not applicable	1	1	Not applicable	0	2	2	2
Bashari et al	2	2	2	2	2	1	2	2	2	Not applicable	2	2	2	0	2	2	2
Gamaoun	2	2	2	1	0	1	2	2	2	Not applicable	2	2	2	0	2	0	2
Hagens et al	2	2	2	1	2	2	2	2	2	2	2	1	2	2	2	2	2
Jit et al	2	2	2	2	2	2	2	2	2	Not applicable	2	2	2	2	2	2	2
Khatibi et al	2	2	2	2	2	2	2	2	2	2	2	1	2	1	2	2	2
Khiari et al	2	2	2	0	0	2	2	2	2	Not applicable	2	2	2	0	2	2	2
Laraj et al	2	2	2	2	2	2	2	2	2	2	2	2	2	2	2	2	2
Messoudi et al	2	2	2	2	2	2	2	2	2	Not applicable	2	2	2	2	2	2	2
Rosettie et al	2	2	2	1	1	2	2	2	2	Not applicable	2	2	2	2	2	2	2
Sargazi et al	2	2	2	2	1	2	Not applicable	2	2	Not applicable	2	2	Not applicable	1	2	2	2

Yaghoubi et al	2	2	2	2	2	2	2	2	2	Not applicable	2	2	2	2	2	2	2
Ezoji et al	2	2	2	2	1	1	2	2	2	2	2	2	2	1	2	2	2
Lagoubi et al	2	2	2	2	1	2	2	2	2	2	2	2	2	0	2	2	2
Pugh et al	2	2	2	2	2	1	2	2	2	2	2	2	2	0	2	2	2
Sevilla et a	2	2	2	2	2	2	2	2	2	2	2	2	2	2	2	2	2
Sibak et al	2	2	2	2	2	2	2	2	2	Not applicable	2	2	2	0	2	2	2
Azad et al	2	2	2	2	2	2	2	2	2	Not applicable	2	2	2	0	2	2	2
Debellut 2020	2	2	2	2	1	1	2	2	2	2	2	2	2	0	2	2	2
Debellut 2021	2	2	2	2	1	2	2	2	2	2	1	2	2	2	2	2	2
Debellut 2022	2	2	2	2	1	1	2	2	1	0	2	2	2	2	2	2	2
Javanbakht et al	2	2	2	2	2	1	2	2	2	2	2	2	2	1	2	2	2
Mohy et al	2	2	2	2	0	2	2	2	2	2	2	2	2	0	2	2	2
Mousavi Jarrahi et al	2	2	2	2	1	1	2	2	2	2	2	2	2	0	2	2	2
Paternina-Caicedo et al	2	2	2	2	2	2	2	2	2	Not applicable	2	2	2	2	2	2	2

Parameters: P1: Title identifies study as economic evaluation; P2: Structured abstract provided; P3: Background/objectives clearly stated; P4: Perspective stated; P5: Time horizon stated & justified; P6: Discount rate reported & justified; P7: Model structure described (diagram if relevant); P8: Modelling assumptions described; P9: Effectiveness evidence sources described; P10: Preference-based outcomes described (if used); P11: Cost/resource estimation methods described; P12: Currency, price year & conversion stated; P13: Incremental results clearly reported (ICERs etc.); P14: Heterogeneity assessed or discussed; P15: Limitations discussed; P16: Funding source declared; P17: Conflicts of interest declared
